# Uptake of Upconverting Nanoparticles by Breast Cancer Cells: Surface Coating versus the Protein Corona

**DOI:** 10.1021/acsami.1c10618

**Published:** 2021-08-11

**Authors:** Evelina Voronovic, Artiom Skripka, Greta Jarockyte, Marija Ger, Dalius Kuciauskas, Algirdas Kaupinis, Mindaugas Valius, Ricardas Rotomskis, Fiorenzo Vetrone, Vitalijus Karabanovas

**Affiliations:** † Biomedical Physics Laboratory of National Cancer Institute, Baublio 3B, LT-08406 Vilnius, Lithuania; ‡ Life Sciences Center, Vilnius University, Sauletekio av. 7, LT-10257 Vilnius, Lithuania; § Department of Chemistry and Bioengineering, Vilnius Gediminas Technical University, Sauletekio av. 11, LT-10223 Vilnius, Lithuania; ∥ Centre Énergie, Matériaux et Télécommunications, Institut National de la Recherche Scientifique, Université du Québec, 1650 Boul. Lionel-Boulet, Varennes, Quebec J3X 1S2, Canada; ⊥ Institute of Biochemistry, Life Sciences Center, Vilnius University, Sauletekio av. 7, LT-10257 Vilnius, Lithuania; # Biophotonics Group of Laser Research Centre, Vilnius University, Sauletekio av. 9, LT-10222 Vilnius, Lithuania

**Keywords:** lithium yttrium fluoride (LiYF_4_), rare-earth-doped nanoparticles, protein corona, cellular uptake, endocytosis, upconversion

## Abstract

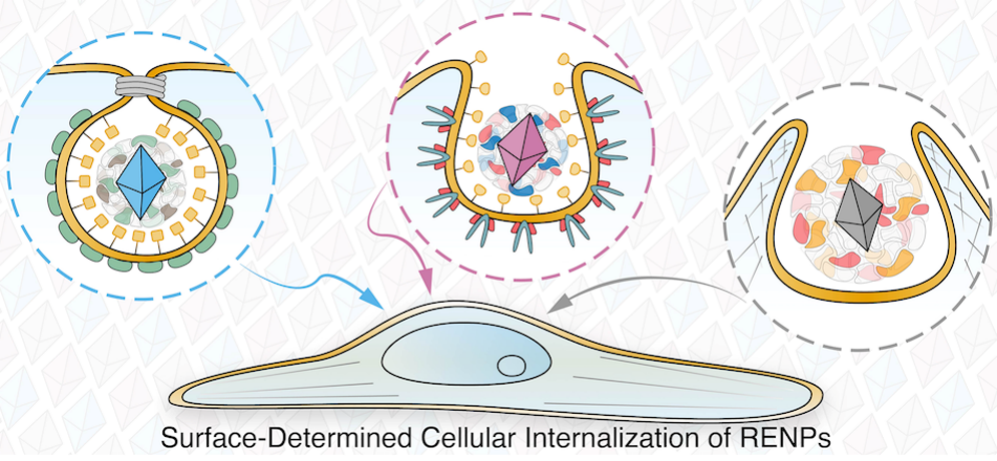

Fluorophores with multifunctional properties known as rare-earth-doped nanoparticles (RENPs) are promising candidates for bioimaging, therapy, and drug delivery. When applied *in vivo*, these nanoparticles (NPs) have to retain long blood-circulation time, bypass elimination by phagocytic cells, and successfully arrive at the target area. Usually, NPs in a biological medium are exposed to proteins, which form the so-called “protein corona” (PC) around the NPs and influence their targeted delivery and accumulation in cells and tissues. Different surface coatings change the PC size and composition, subsequently deciding the fate of the NPs. Thus, detailed studies on the PC are of utmost importance to determine the most suitable NP surface modification for biomedical use. When it comes to RENPs, these studies are particularly scarce. Here, we investigate the PC composition and its impact on the cellular uptake of citrate-, SiO_2_-, and phospholipid micelle-coated RENPs (LiYF_4_:Yb^3+^,Tm^3+^). We observed that the PC of citrate- and phospholipid-coated RENPs is relatively stable and similar in the adsorbed protein composition, while the PC of SiO_2_-coated RENPs is larger and highly dynamic. Moreover, biocompatibility, accumulation, and cytotoxicity of various RENPs in cancer cells have been evaluated. On the basis of the cellular imaging, supported by the inhibition studies, it was revealed that RENPs are internalized by endocytosis and that specific endocytic routes are PC composition dependent. Overall, these results are essential to fill the gaps in the fundamental understanding of the nano-biointeractions of RENPs, pertinent for their envisioned application in biomedicine.

## Introduction

Photoluminescent nanoparticles (NPs) are actively studied as building blocks in multifunctional nanotools for a plethora of biomedical applications. These nanomaterials differ in size, shape, composition, and function; thus, rationally engineered NPs act as probes for biosensing, optical imaging, therapy, and nanoscale thermometry, to name a few.^1−4^ In the context of *in vitro*/*in vivo* use of NPs, successful NP entry into the cell and accumulation of NPs at the targeted site becomes vital. It is imperative to have knowledge of the NP behavior in a biological environment, interaction with cells, as well as the fate of the NPs once inside the cell.^5−7^ Furthermore, for NPs to be effectively translated to the clinic, fundamental studies of their photochemical properties must be complimented with research on their biological behavior and cytotoxicity.^3,7−10^

When NPs are introduced into complex biological fluids, the constituents of blood or the cell-culture medium change their superficial representation,^11,12^ forming the so-called protein corona (PC). The PC is dynamic and unique for each new nanomaterial, and depends on NP shape, size, surface charge, and composition of the biological fluid in question.^6,12,13^ Typically, the NP–PC complex interacts with the cell by adsorption on the cell membrane, following engulfment and accumulation in the cytoplasm via the endocytosis. The precise mechanism of endocytosis is determined by the cell line, morphology, and charge of the NPs, as well as the features or composition of the surrounding PC.^12,14,15^ The latter influences the cellular uptake mechanism^6^ by altering the surface composition and the size of the NPs.^16^ Thus, knowledge of the protein profile on the NP surface and control over PC formation is essential for targeted drug delivery and for the ensuing clinical success of the NPs.^12,16^ In some cases, the PC prevents targeted delivery of NPs and their cargo, which can result in cellular uptake inhibition. Furthermore, opsonized NPs cannot escape uptake by the mononuclear phagocyte system (MPS).^17^ Previous approaches to reduce protein binding on the surface of the NPs involved functionalization with poly(ethylene glycol) (PEG), dextran, or poly(vinylpyrrolidone) (PVP),^18,19^ which reduced protein binding and prolonged blood-circulation time.^20^ Yet, PC formation is unavoidable and occurs during the entire path of the NP toward the target site.^12^ For this reason, both the biocompatibility of the NPs and their PC composition should be considered and thoroughly investigated, especially for relatively new classes of NPs whose nano-biointeractions tend to be underrepresented early on.^9,12,21^

Rare-earth-doped NPs (RENPs) are a promising new class of photoluminescent nanomaterials^1^ whose primary advantage over more traditional NPs lies in their ability to convert low energy photons into high energy ones via a multiphoton process known as upconversion (UC), typically transforming near-infrared (NIR) excitation to UV/visible emission.^22^ Excitation by NIR radiation within the biological imaging window (750–950 nm), where light attenuation by absorption and scattering is reduced, means that RENPs can be phototriggered at greater tissue depths, providing high-energy photons, necessary to drive therapeutic processes, *in situ*.^1,23^ Moreover, the photoluminescence emission wavelength of RENPs does not strictly depend on their crystal dimensions, as in the case of quantum dots.^24,25^ Through variation of their internal architecture (choice of dopants, multishelling, *etc.*), it is possible to synthesize RENPs of the same size and shape, rationally tailored to the specific *in vivo* bioimaging or the therapeutic task at hand.^26^ Hence, insights from certain RENPs, scrutinized under the lens of biological interactions, can be successfully extrapolated and applied to other RENPs of the same morphology and size, eliminating the need for laborious and unnecessary repetition of studies that could otherwise delay their clinical application.

Here, we investigated the unspecific cellular internalization and PC formation for LiYF_4_:Yb^3+^,Tm^3+^ RENPs. These RENPs are well regarded for their ability to convert NIR excitation into UV emission and are ultimately harnessed for controlled drug delivery.^8,27−33^ Since the surface of RENPs plays a lead role in determining the PC composition, we studied RENPs functionalized with citrate, phospholipid, and SiO_2_ (silica) coatings, which are among the commonly used surface modifications in RENP research. For our study we selected two cell lines, MDA-MB-231 and MCF-7, as breast cancer model systems. MCF-7 cells are luminal breast cancer cells while MDA-MB-231 are more aggressive, exhibiting cancer stem-like properties and are triple-negative basal-type cancer cells. By investigating the PC composition and size around citrate-, silica-, and phospholipid-coated RENPs, we could differentially pinpoint different aspects that impact cellular internalization efficiency and pathways of RENPs. We believe that the knowledge regarding the PC on the surface of the RENPs, acquired through these studies, can make headway into the better and safer use of these NPs in nanomedicine.

## Results

### Characterization of RENPs

**Figure 1:**
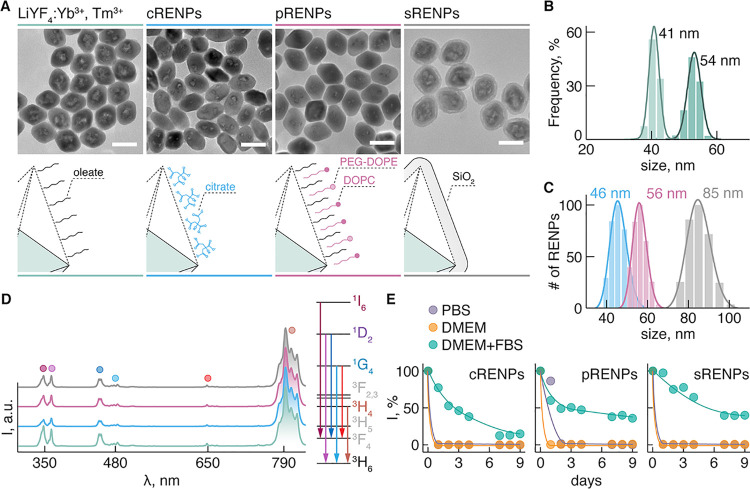
Structural and spectral characterization of RENPs. (A) Transmission electron microscopy (TEM) images and schematic representation of LiYF_4_:Yb^3+^,Tm^3+^ RENPs bearing different surface coatings: cRENPs—citrate, pRENPs—phospholipids, and sRENPs—SiO_2_. Color coding for different coatings is maintained across other graphs in the figure. (B) Size distribution of the synthesized oleate-capped LiYF_4_:Yb^3+^,Tm^3+^ RENPs along their major and minor axes. (C) Average hydrodynamic size of cRENPs, pRENPs, and sRENPs dispersed in distilled water at a 0.5 mg/mL concentration. (D) Representative upconversion emission spectra of RENPs in hexane and in water. Dots above each major emission band are color coded to the radiative transition shown on the right. (E) Colloidal stability of cRENPs, pRENPs, and sRENPs dispersed in PBS, DMEM, and in DMEM supplemented with 10% of FBS over a period of 9 days. Lines are guides to the eye.

We prepared oleate-capped LiYF_4_:25 mol% Yb^3+^, 0.5 mol% Tm^3+^ RENPs via the thermal decomposition synthesis in organic media, and subsequently rendered the RENPs water dispersible by surface modification with citrate ligands (cRENPs), phospholipids (pRENPs), or silica (sRENPs) (Figure 1A). The parent oleate-capped RENPs featured a bipyramidal morphology and were approximately 54 nm × 41 nm in size along their major and minor axes, respectively (Figure 1B), possessing a pure tetragonal phase identified by X-ray powder diffraction (XRD) analysis (Figure S2). Moreover, the success of the surface modification (*i.e.*, the presence of the different surface coatings) was confirmed by Fourier transform infrared (FTIR) measurements (Figure S3). It should be noted that both morphology and size of the parent RENPs were preserved after each surface modification (Figure 1A). Since the colloidal stability and size of the RENPs strongly depend on the biological medium in which they are dispersed, we measured their hydrodynamic size and zeta potential (ζ). The hydrodynamic size of RENPs in distilled water, was found to be around 46, 56, and 85 nm with polydispersity indices (PDI) of 0.23, 0.16, and 0.14 for cRENPs, pRENPs, and sRENPs, respectively (Figure 1C). The ζ potential of cRENPs was −25.1 mV, that of pRENPs −10.7 mV, and sRENPs presented a value of −44.0 mV (pH 6.2). Under 980 nm excitation, Tm^3+^ upconverted photoluminescence was observed from all RENPs, where the major emission bands were centered around 340 (^1^I_6_ → ^3^F_4_), 360 (^1^D_2_ → ^3^H_6_), 450 (^1^D_2_ → ^3^F_4_), 480 (^1^G_4_ → ^3^H_6_), 510 (^1^D_2_ → ^3^H_5_), 660 (^1^G_4_ → ^3^F_4_), and 790 nm (^3^H_4_ → ^3^H_6_) (Figure 1D). The wide range of available emissions (from UV to NIR) uniquely position Yb^3+^/Tm^3+^-doped RENPs as prominent therapeutic and diagnostic agents, especially when it comes to applications demanding high-energy photons to drive photochemical reactions. Prior to *in vitro* studies, we measured the transient colloidal stability of RENPs bearing different surface coatings. The RENPs were colloidally stable in distilled water; however, for application in a biological context, colloidal stability in media mimicking the physiological environment is more important. Figure 1E shows the time-dependent behavior of RENPs in different media (phosphate-buffered saline—PBS, Dulbecco’s modified Eagle medium—DMEM, and DMEM with 10% fetal bovine serum—DMEM + FBS). The colloidal stability of RENPs in the different media was measured as the change of upconversion emission intensity of the RENPs over 216 h (9 days) (see Supporting Information for more details). It was found that the proteins in FBS (Figure 1E) play a significant role in preventing RENPs from aggregation and ensure colloidal stability of the RENPs when exposed to physiological fluids (PBS, DMEM). Furthermore, alterations in surface identity of RENPs, as an early indication of PC formation, could be evidenced from their reduced ζ potential when dispersed in DMEM + FBS; values of −10.1, −6.7, and −7.5 mV were measured for cRENPs, pRENPs, and sRENPs, respectively.

### Biocompatibility and Accumulation of RENPs in Cancer Cells

**Figure 2:**
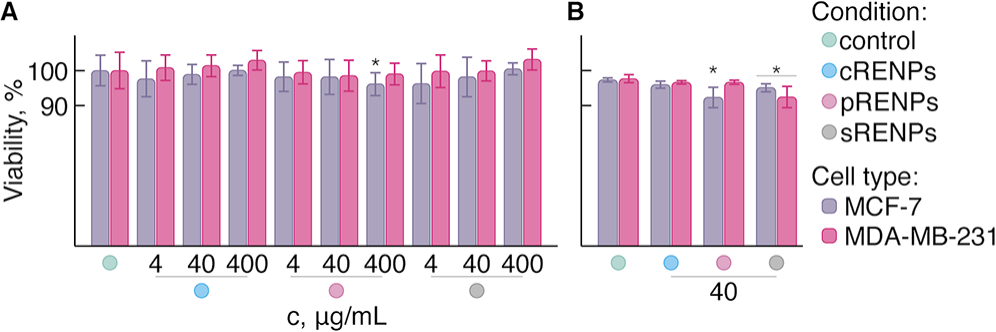
Viability of two breast cancer cell lines, MCF-7 and MDA-MB-231, incubated with cRENPs, pRENPs, and sRENPs. (A) LDH assay at varying concentrations of RENPs: 4, 40, and 400 μg/mL and (B) direct count of the viable cells with the mammalian automatic cell counter ADAM-MC (at a set RENP concentration of 40 μg/mL). Values of 100% indicate total cellular viability (control data). * indicates significant differences compared to the nontreated cells (control) (*p* ≤ 0.05). Viability values were calculated as mean ± standard deviation (*N* = 3, *n* = 6).

To be certain of the biocompatibility of the various RENPs, we evaluated their cytotoxicity by two independent methods: lactate dehydrogenase (LDH) assay (colorimetric assay for cytotoxicity) and direct automatic counting of viable cells (determination of cell viability). Cytotoxicity assays usually provide insight into the toxicity of a material (RENPs) to the cells, whereas, cell viability assays reveal how many cells are viable after exposure to RENPs. In the case of the LDH assay (Figure 2A), different RENP concentrations were used (4, 40, and 400 μg/mL), while viable cell counting was performed on cells incubated with 40 μg/mL of RENPs (Figure 2B). After 24 h of incubation with RENPs, no statistically significant effect on the cell viability was observed from the LDH assay for any of the differently coated RENPs or their concentration with respect to the control. Similarly, results obtained from the ADAM-MC mammalian cell counter (Figure 2B) also showed RENPs to be biocompatible. This congruence between the results obtained with the two assays independently ascertained the biocompatibility of the studied RENPs, and therefore, were deemed suitable for further investigations.

**Figure 3:**
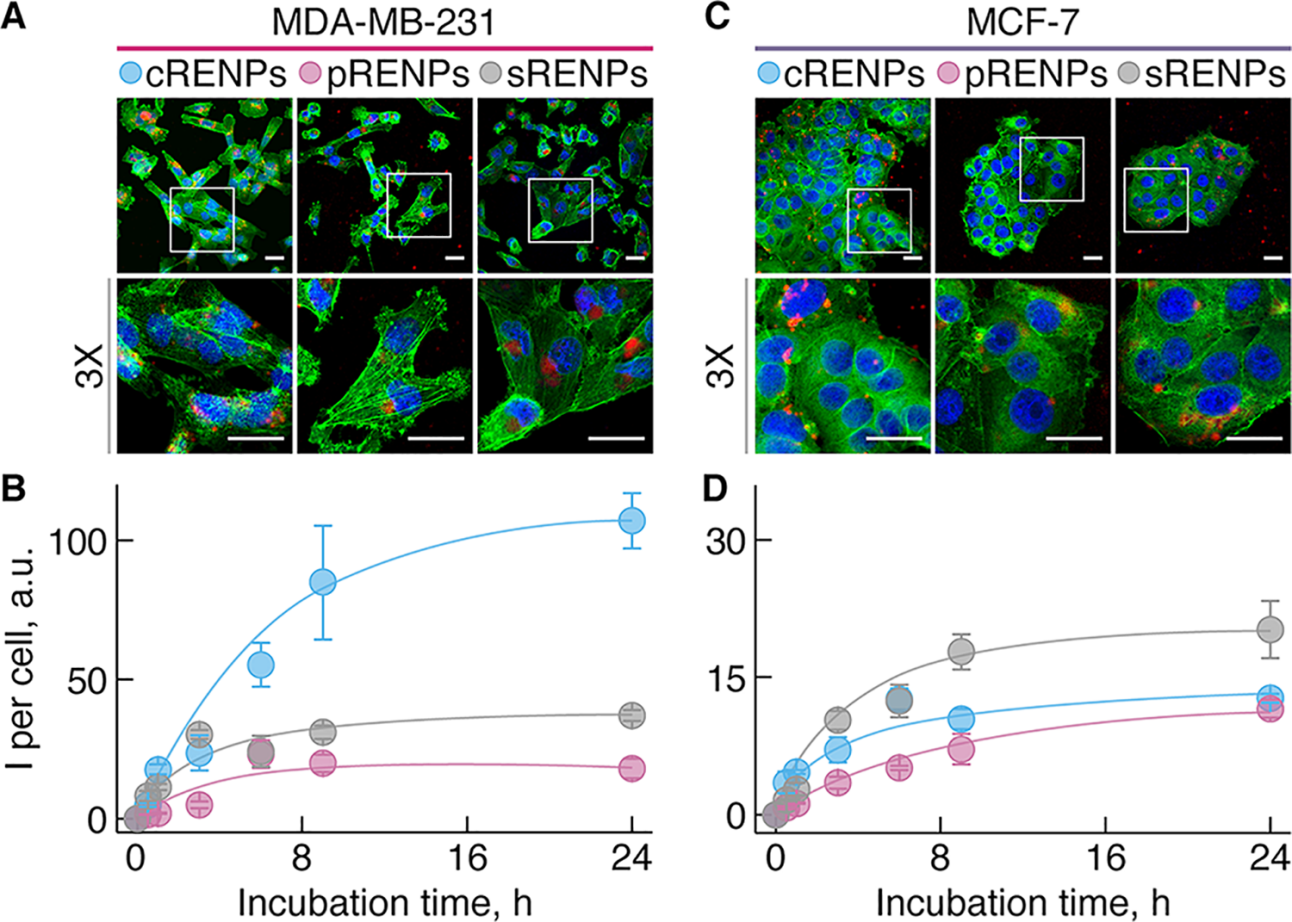
Comparison of the cellular uptake of RENPs by MDA-MB-231 (A, B) and MCF-7 (C, D) breast cancer cells. (A, C) Confocal fluorescence microscopy images of cells after 24 h of incubation with RENPs. Upconversion emission signal obtained under 980 nm excitation is represented by the color red in all cases. Cell nuclei were stained with Hoechst (blue) (λ_ex_ = 404 nm) and F-actin was stained with Phalloidin-Alexa 488 (green) (λ_ex_ = 488 nm). Representative cell images are shown in a wide-field and 3× zoom view. Scale bars in all images are 25 μm. (B, D) Accumulation dynamics of RENPs, assessed by the average upconversion emission intensity per cell. Intensity values were calculated as mean ± standard deviation (*N* = 3, *n* = 3).

Laser scanning confocal microscopy (LSCM) allowed to assess the uptake of RENPs with different coatings in cancer cells after various exposure times (1, 3, 6, and 24 h). LSCM results at shorter incubation times (1, 3, 6 h) are presented in Figure S4, while images obtained after 24 h of incubation are shown in Figure 3, as representative of the RENPs’ accumulation. Already after 1 h of incubation, RENPs are situated in the vesicular structures, likely endosomes, and are located in the cytoplasm of the cell (Figure S4). Prolonging their exposure time to cells, after 3 and 6 h incubation, vesicles stored more RENPs than after shorter incubation times. The quantity of RENPs in vesicles is directly proportional to the emission intensity of the RENPs, hence more RENPs in the vesicles are reflected by the greater acquired photoluminescence signal. Overlapping the RENP emission with nuclei and cytoskeletal fluorescence clearly demonstrates that the RENPs are in the cytoplasm rather than inside the nucleus. We suppose that differently coated RENPs were trapped in vesicles, which were located near the nuclei of the cells (Figure 3A).

The main qualitative observation between RENPs bearing different coatings was the quantity of RENPs in vesicles. Visually, uptake of RENPs in MDA-MB-231 cells was much greater compared to MCF-7 cells. The majority of MDA-MB-231 cells had accumulated RENPs after 24 h incubation (Figure 3A), whereas only a few MCF-7 cells had accumulated any RENPs (Figure 3C) in the same time period. In fact, the low uptake dynamics of RENPs in MCF-7 cells (Figure 3C) made it much more difficult to identify the vesicular structures. LSCM images of MDA-MB-231 cells indicated that uptake of RENPs with different coatings was also dissimilar: accumulation of cRENPs was highest while that of pRENPs was the lowest.

To prove these qualitative observations, we performed a more accurate assessment of the RENP accumulation dynamics by measuring their net intensity within the cells after 0.5, 1, 3, 6, 9, and 24 h incubation (see the Experimental Section for more details). Cellular uptake was calculated as average emission intensity per cell (Figure 3B,D). The RENP emission intensity in both cell lines grew until a plateau was reached, at around 9 h of incubation. In the MDA-MB-231 cells, the RENP emission was notably higher than in MCF-7 cells, attesting to the fact that the former cells tend to accumulate RENPs better than the latter ones. As can be seen from the uptake dynamic curves, cRENPs are taken up by MDA-MB-231 cells significantly more than RENPs with the two other coatings. After 24 h of incubation, the average emission intensity of cRENPs in MDA-MB-231 cells was 6 times higher than that of pRENPs and 3 times higher than sRENPs. In the case of MCF-7 cells, the highest emission intensity per cell was detected after incubation with sRENPs, and the signal intensity was about 2 times higher than that of cRENPs and pRENPs.

### Effect of Inhibitors on Endocytic Pathways of RENPs

To gain further insight into the RENP internalization pathways within the cancer cell lines (MDA-MB-231 and MCF-7), we systematically examined the accumulation of RENPs inside these cells, treated with various inhibitors against specific endocytic mechanisms (for experimental details, please refer to the Supporting Information). We have used the lipid raft/caveolin-mediated endocytosis (CVME) inhibitor nystatin (Nys), clathrin-mediated endocytosis (CME) inhibitor chlorpromazine (Chlor), the microtubule assembly/disassembly dynamics disrupter nocodazole (Noc), which inhibits CME, and 5-(N-ethyl-N-isopropyl)amiloride (EIPA) as a macropinocytosis blocker. RENPs were also incubated with cells at 4 °C to observe if an energy-dependent internalization process was present.^5,34^ Concentrations of inhibitors used in this study are listed in Table S1 in the Experimental section.

Both cell lines were preincubated with different inhibitors at 37 °C for 1 h prior to incubation with RENPs for 3 h. Additionally, some cells were exposed to low-temperature conditions (4 °C). By lowering the temperature, energy-dependent endocytosis is inhibited and as a result should significantly reduce uptake of all RENPs in both cell lines used.

**Figure 4:**
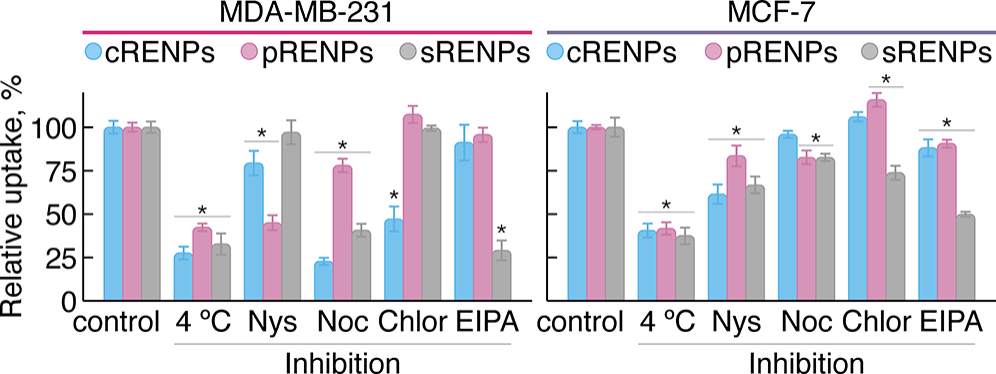
Effect of internalization pathway inhibition on the uptake of differently coated RENPs by MDA-MB-231 (left) and MCF-7 (right) breast cancer cells. Inhibitor untreated cells, incubated with RENPs at 37 °C, were used as a control. * indicates significant differences compared to the control (*p* ≤ 0.05). Uptake values were calculated as mean ± standard deviation (*N* = 3, *n* = 3).

As can be seen in Figure 4, endocytosis inhibition clearly depends on the surface coating of the RENPs and the cell line. Nys inhibited the uptake of pRENPs (emission intensity, herein relative uptake, of RENPs inside the cells was reduced to 45% compared to the control) in MDA-MB-231 cells and showed little to no inhibition for cRENPs (79%) and sRENPs (97%), respectively. Noc treatment inhibited uptake of cRENPs (23%), sRENPs (41%), and least that of pRENPs (78%). Chlor had the same effect on the uptake of cRENPs as Noc, reducing the internalization rate to 47%. Furthermore, Chlor had no effect on sRENPs and pRENPs compared with control samples. EIPA presented with the inhibitory effect only on the uptake of the sRENPs, reducing it to 29%. In the case of the MCF-7 cell line, cRENP and sRENP internalization efficiency was reduced by Nys to 61 and 67%, respectively. Noc inhibition reduced the uptake of pRENPs and sRENPs to 83%; however, showed no effect on the internalization of cRENPs in MCF-7 cells. Chlor treatment resulted in enhanced internalization of cRENPs and pRENPs but a slight reduction in the uptake of sRENPs (74%). EIPA showed a slight reduction in the uptake of cRENPs (88%) and pRENPs (91%), while internalization of sRENPs was reduced to 50%.

**Figure 5:**
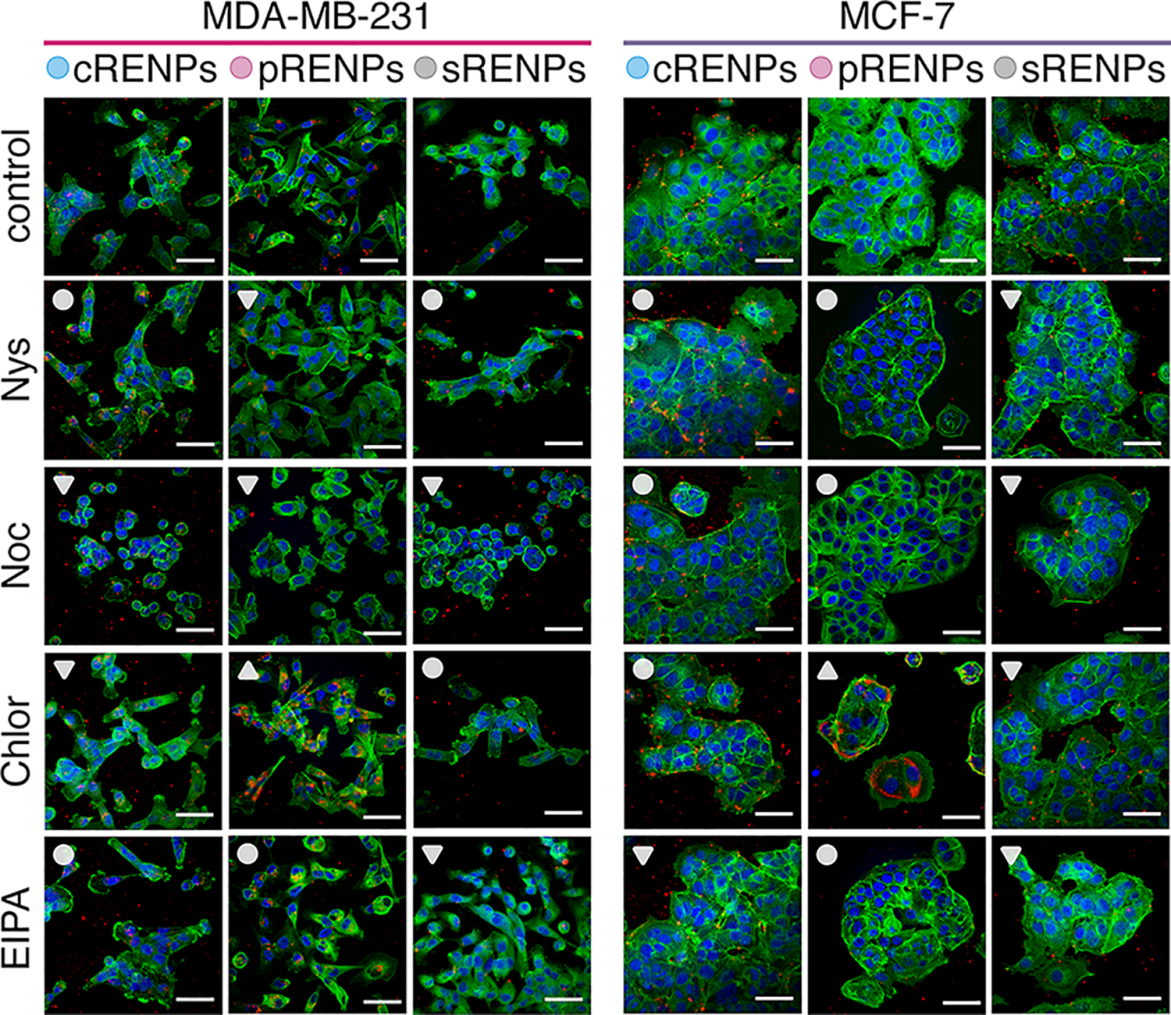
LSCM images of RENP accumulation in MDA-MB-231 and MCF-7 breast cancer cells after 1 h of treatment with inhibitors and 3 h of incubation with RENPs. Control refers to noninhibited cells incubated with RENPs for 3 h. Upconversion emission signal, obtained under 980 nm excitation, is represented by the color red in all cases. Cell nuclei were stained with Hoechst (blue) (λ_ex_ = 404 nm) and F-actin was stained with Phalloidin-Alexa 488 (green) (λ_ex_ = 488 nm). Symbols △,▽, and ○ are used to represent increase, decrease, and no change in the observed upconversion emission due to inhibition, respectively. Scale bars are 50 μm.

To confirm the inhibition effect on the internalization of RENPs inside the cells, LSCM imaging was performed. Control cells were incubated with RENPs for 3 h without any additional treatment, while experimental cells were treated with inhibitors for 1 h before incubation with RENPs. As can be seen in Figure 5, the results obtained in MDA-MB-231 cells are in good agreement with the data presented in Figure 4. The uptake of cRENPs in MDA-MB-231 cells was inhibited with Noc and Chlor, while Nys and EIPA had no effect on the internalization of cRENPs. The internalization of pRENPs in MDA-MB-231 cell was inhibited with Nys and Noc, the internalization of sRENPs was inhibited with Noc and EIPA, while Nys and Chlor had no impact. On the other hand, only EIPA inhibited the accumulation of cRENPs in MCF-7 cells whereas other inhibitors showed no discernable effect. All of the investigated inhibitors decreased the accumulation of sRENPs in MCF-7 cells. In both MDA-MB-231 and MCF-7 cells, Chlor increased uptake of pRENPs, while EIPA had no effect on their accumulation. It should be noted that accumulation of RENPs in MCF-7 cells after 3 h of incubation was relatively weak even in control samples and most of the RENPs attached only to the membrane of the cells (as seen from the LSCM pictures in Figure 5). Thus, it is difficult to detect uptake differences between control and inhibitor treated cells. We, therefore suggest that any final conclusions regarding the effect of inhibitors as well as results of the accumulation of RENPs should only be arrived at from data obtained with more sensitive instrumentation, such as a spectrometer (Figure 4), rather than from confocal fluorescence microscopy images alone.

### Protein Corona of RENPs

**Figure 6:**
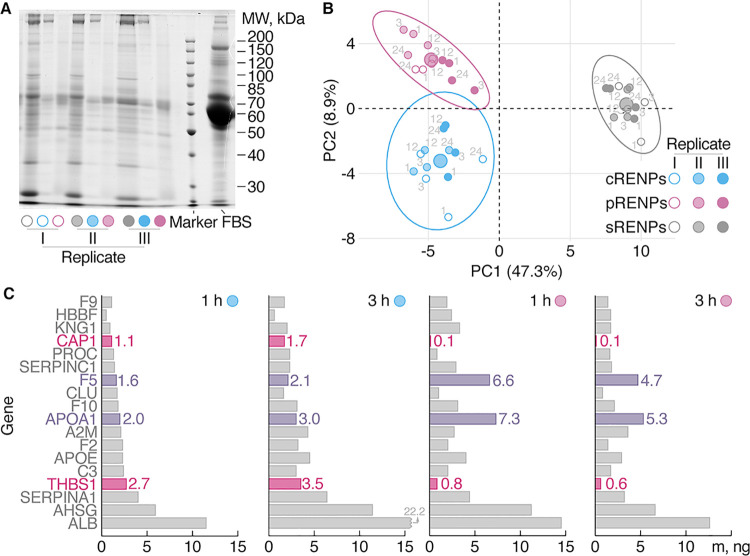
RENPs with different coatings form different PCs. (A) Gel electrophoresis of proteins eluted from RENPs incubated with the FBS-containing DMEM (incubation time 24 h, *N* = 3) compared to FBS (1 μL). (B) Clustering of RENPs incubated with serum-containing media based on the PC composition identified by mass spectrometry (incubation time of 1, 3, 12, 24 h is indicated next to each data point; *N* = 3). (C) Proportions of the most abundant proteins in the PC of cRENPs and pRENPs after 1 or 3 h of incubation in DMEM with FBS. Highlighted bars showcase the most abundant proteins on the surface of cRENPs and pRENPs, relative to each other.

The composition of the PC that forms around the RENPs with different coatings was assessed and compared by means of proteomic analysis. First, the total amount of protein bound to cRENPs, pRENPs, or sRENPs and the reproducibility of the PC formation were assessed by gel electrophoresis after 24 h of incubation in an FBS-containing DMEM (Figure 6A). Notably, sRENPs bound ∼5-fold more total protein compared to cRENPs or pRENPs. The protein electrophoresis pattern demonstrated that RENPs with different coatings bind coating-specific sets of serum proteins.

The proteins eluted from the RENPs after various incubation times were digested with trypsin, then identified and quantified via high-definition liquid chromatography–mass spectrometry (LC–MS; Supplemental Table S1). Overall, 67, 63, and 86 proteins were detected on the surfaces of cRENPs, pRENPs, or sRENPs, respectively. Around the cRENPs, the most abundant proteins were serum albumin, α-2-HS-glycoprotein, and α-1-antiproteinase. In the case of pRENPs, the most abundant proteins were similarly serum albumin and α-2-HS-glycoprotein along with apolipoprotein A-I, while sRENPs showcased the presence of serum albumin, A2M, as well as the coagulation factor V (F5). Protein identification results showed the contrast between the PCs for all three differently coated RENPs. Principal component analysis (PCA) (Figure 6B) helps to visualize the differences in the composition of the PC between the three surface coatings and the results are presented as separate clusters in a graph, showcasing differences in PC components for every RENP surface coating. The composition of pRENP and cRENP PC was stable in time up to 24 h. The PC of sRENPs, however, underwent a more prominent change compared to other RENPs. In particular, the amount of F5 and prothrombin, as well as of α-2-HS-glycoprotein increased considerably, while the amount of gelsolin and that of the elements of the complement system (factors H, C3) decreased (Supplemental Table S1). At the outset, all three RENPs form a different PC of serum proteins, which, in turn, determines their subsequent cellular uptake.

We further compared the PC composition between cRENPs, pRENPs, and sRENPs in search for the potential cause of the different accumulation dynamics of RENPs in MDA-MB-231 and MCF-7 cell lines. Proteomes at 1 and 3 h of RENP incubation in an FBS-containing medium (Supplemental Table S2) were selected for detailed analysis before the uptake of RENPs reaches a plateau. The total amount of protein in the PC of cRENPs and pRENPs was similar after 1 and 3 h of incubation time. Approximately, 20% of the PC components (14 proteins) differed in the concentration between the two types of RENPs after 1 h of incubation but only 8 proteins differed at both 1 and 3 h time points. Of these eight proteins, two proteins were overrepresented in the PC of cRENPs: THBS1 and adenylyl cyclase-associated protein 1 (CAP1). In the case of the PC for pRENPs, six proteins could potentially determine the difference in their cellular uptake (Figure 6C). As mentioned above, sRENPs bound ∼5-fold more of total protein than other coatings. Such PC of sRENPs may be due to a larger surface area and a negative surface charge compared to other counterparts. Overall, 51 proteins showed differences in the quantity between the sRENPs and pRENPs at 1 h and 44 at both time points (1 and 3 h). Of these different proteins, 46 were overrepresented in the sRENPs PC after 1 h of incubation and 39 at both, 1 and 3 h, time points. The analysis of the biological pathways enrichment with the Enrichr tool showed that proteins involved in blood clotting and the complement system dominated in the PC of sRENPs (Supplemental Table S3). Proteins unique for each PC (CAP1, THBS1 for cRENPs; F5, ApoA1 for pRENPs; A2M, F5 for sRENPs) are the most likely candidates responsible for difference in cellular uptake.

To elucidate the factors underlying the difference in the uptake of RENPs between the analyzed cell lines, we performed a comparative proteomic analysis of the cell surface proteome of MCF-7 and MDA-MB-231 cells. Cell surface proteins were enriched via biotin labeling and identified by high-definition LC–MS (Supplemental Table S4). Unbiotinylated cells were used as a control for unspecific protein binding. Overall, 399 proteins were identified in total, and of these, 99 proteins were unique to or significantly increased in MCF-7 cells, while 157 proteins were unique to or significantly increased in MDA-MB-231 cells. The surface proteins of both cell lines were grouped into categories using the Gene Ontology (GO) cellular component classification. The top 10 categories for MCF-7 and MDA-MB-231 cells are presented in Table S2 and Table S3, respectively. The analysis of both data sets confirms the high enrichment of plasma membrane proteins. The MCF-7 cell membrane fraction is characterized mostly by a high level of the cytoskeleton and of intermediate filament proteins. Conversely, the MDA-MB-231 cells exhibit enrichment in cellular transport and endocytotic proteins, especially clathrin-coated vesicle membrane components (Figure S5). These differences in the plasma membrane and related proteins of both cell lines correlate with the experimental differences in the uptake of RENPs: Chlor and microtubule assembly disrupter Noc that both target CME decreased the uptake of RENPs in MDA-MB-231 cells more efficiently than in MCF-7 cells (Figures 4 and 5).

**Figure 7:**
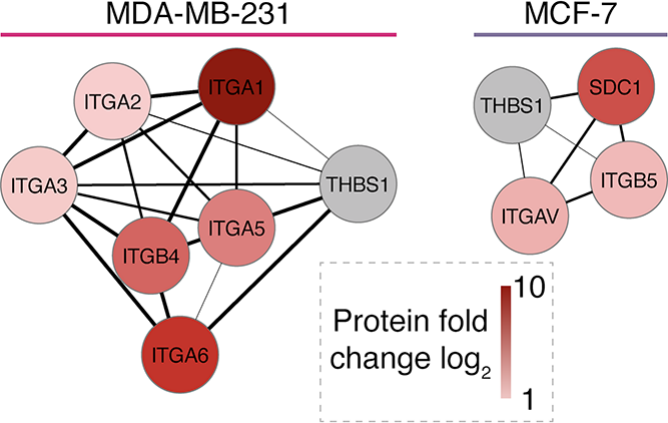
Analysis of the cell surface proteome of MCF-7 and MDA-MB-231 cells. Proteins from MCF-7 or MDA-MB-231 surface proteome potentially interacting with THBS1. The interaction network was visualized with Cytoscape, using data retrieved from the STRING database. The relative widths of the connection lines represent the combined score of protein–protein interaction; the intensity of the node color is proportional to the relative amount of the protein in the enriched cell surface proteome

Finally, we combined the results of both proteomic analyses aiming to predict the proteins on the surface of the cells capable of interacting with the specific proteins of the PC of RENPs. Such interactions could be responsible for the different uptakes of cRENPs and pRENPs. Proteins THBS1 and CAP1 that were significantly increased in cRENPs and correlated with the intensive uptake of cRENPs compared to pRENPs were selected for the analysis. Protein–protein interaction data were retrieved from the STRING database. In the case of CAP1, no potential interacting proteins were found either in the MCF-7 or in the MDA-MB-231 cell surface proteome. On the other hand, we have identified three interacting proteins in MCF-7 cells for THBS1: syndecan 1, two members of the integrin family (ITGAV and ITGB5) and six interacting proteins in the MDA-MB-231 proteome (all members of the integrin family) (Figure 7). The larger variety and the higher presence of THBS1-interacting proteins in the MDA-MB-231 cell surface may explain the higher uptake of cRENPs with THBS1-enriched PC.

## Discussion

In general, internalization of NPs by cells can vary depending on several factors: size, shape, charge, surface modification, as well as the cell line itself.^14,35^ Previously, NP sizes around 50 nm were thought to be optimal to minimize the energy used to wrap NPs by the cellular membrane. In fact, rod-like, especially diamond-shaped NPs with aspect ratios around 1.7, exhibited the best internalization and retention inside cells.^8,36^ Both factors, size and morphology, allow to conquer rapid renal filtration and clearance of the NPs via the MPS.^37^ Other studies have shown that rod-like NPs can penetrate and accumulate in cancer tissues more rapidly than spherical ones.^38^ In contrast, Chen et al. recently observed greater accumulation of quasi-spherical RENPs, of around 18 nm in size, as compared to the rod-like shaped RENPs.^39^ It is clear that size and morphology are important, but they alone do not determine the interactions of NPs with biological systems. The cell senses the surface of the NPs, which thus plays a crucial role in the potential uptake of NPs as well as their use in imaging, drug delivery, and therapeutics.^14^

**Figure 8:**
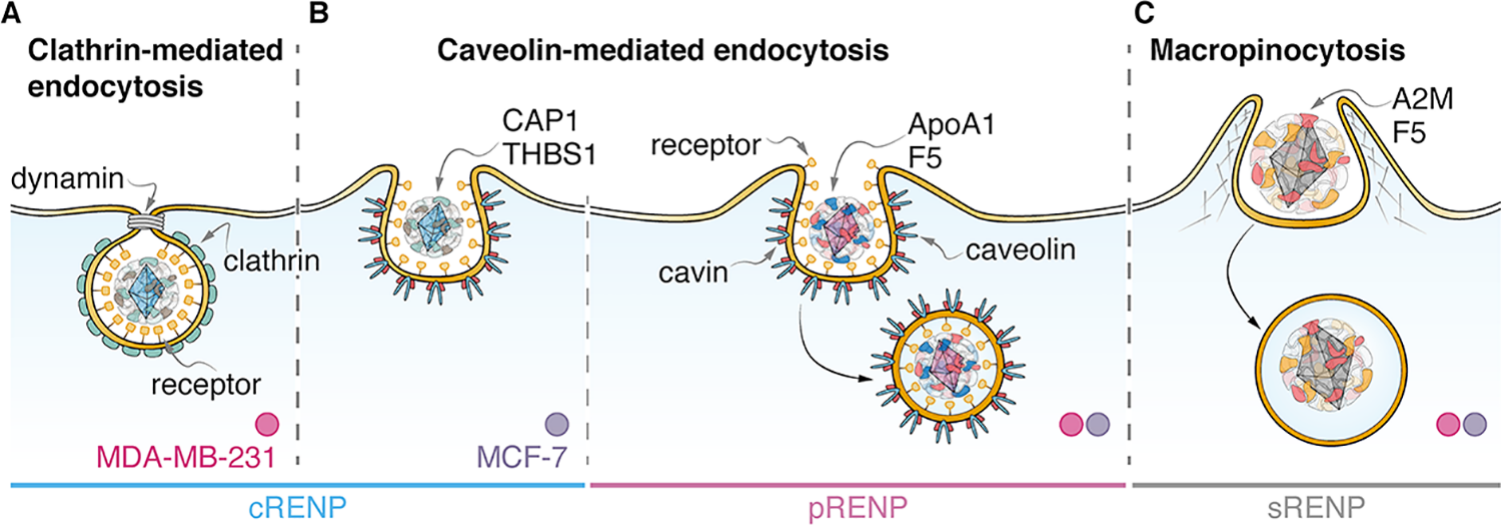
Schematic illustration of internalization pathways of the RENPs in MCF-7 and MDA-MB-231 breast cancer cells following the corresponding distinctive proteins around each RENP. The cRENPs are coated by CAP1 and THBS1 that are responsible for clathrin-mediated endocytosis in MDA-MB-231 cells (A) and caveolin-mediated endocytosis in MCF-7 cells (B). pRENPs have ApoA1 and F5 proteins, which target the caveolin-mediated uptake (B), while sRENPs are covered with distinctive A2M and F5 proteins and are taken up via macropinocytosis in both cell lines (C)

In our study, the LiYF_4_:Yb^3+^,Tm^3+^ RENPs had a bipyramidal morphology (*i.e.*, diamond-like) with an aspect ratio of 1.3 (length of major and minor axes were 54 and 41 nm, respectively, as determined by the TEM) (Figure 1A,B) and were coated with citrate ligands, phospholipids, or silica. We focused on the PC formed around these RENPs once exposed to biological fluids as well as its impact on their biocompatibility and cellular uptake mechanisms. On the basis of these results, we propose a schematic illustration for potential intracellular uptake depending on the cell line and PC around the RENPs (Figure 8).

The PC has a major impact not only on the stability of the RENPs but also on their uptake by cells. When NPs are introduced in a medium containing FBS, protein molecules tend to cover the surface of the NP, which makes it easier to be detected by the cell.^12^ For example, malignant cells tend to accumulate albumin and amino acids as an energy source.^40^ Crucially, the NP coating determines the nature of the proteins that would attach to their surface. For instance, NPs grafted with PEG have reduced formation of the PC,^20^ which generally leads to lower uptake by cells. PEG creates a hydrophilic protective layer around the NP^41^ that results in lower protein binding on the NP surface, cellular uptake reduction, and prolonged NP half-life in blood.^42^ As is demonstrated by our uptake curves (Figure 3), cellular uptake of PEG bearing pRENPs is reduced when compared to other coatings. Moreover, the nature of the cell line also determines the uptake of RENPs (Figure 3). MDA-MB-231 cells tend to accumulate higher amounts of RENPs compared to MCF-7 cells. These differences are due to the fact that MCF-7 cells grow in clusters,^23^ restricting exposure of RENPs to the cells. RENPs accumulate only in the outer cell layer of the colonies, reflected by the poor uptake dynamics.

Usually, the cellular internalization route of the RENPs depends on the specific protein and corresponding receptor binding on the cellular membrane.^14^ To elucidate the specific internalization mechanism of RENPs by cancer cells, we performed endocytosis inhibition studies with five different inhibitors. There are obvious differences in cellular uptake of the various RENPs between MDA-MB-231 and MCF-7 cells after treatment with inhibitors (Figures 4 and 5). We observed that cRENPs enter MDA-MB-231 cells via CME (Figure 4). In most cases, carboxylated NPs with hydrodynamic diameters ranging from 40 to 200 nm are taken up by CME in different cell lines: MDA-MB-231 and MCF-7,^43^ 132N1.^44^ On the other hand, pRENPs appear to enter MDA-MB-231 cells via CVME, which agrees with previous research^8^ where lipid-coated RENPs (with a size of 92 and 53 nm, along their major and minor axes, respectively) entered cells via CVME. Nonetheless, the same authors also claimed that lipid-coated RENPs entered cells via CME, pinpointing the possibility for multiple internalization pathways for the same RENPs. We observed no evidence of CME-based uptake of pRENPs. Our results with sRENPs in MDA-MB-231 and MCF-7 cells correlated with the data of Francia et al., where the uptake pattern of SiO_2_ NPs (having a hydrodynamic size of 50 nm) in HeLa cells was confirmed to be driven by macropinocytosis.^6^ As observed from Figures 4 and 5, for the MCF-7 cell line, all of the inhibitors used in this study have a weaker effect on the endocytosis of RENPs compared with MDA-MB-231 cells. This effect can be due to the tendency of MCF-7 cells to grow in colonies, where only the outer layer of the colony is affected by the inhibitors and is susceptible to contact with the RENPs. In our case, the internalization route for sRENPs in MCF-7 cells is macropinocytosis and CME for cRENPs.

The difference in the composition of the PC plays a major role in the internalization and transport pathways of NPs.^9,12,17^ Therefore, we performed in-depth differential proteomic analysis of the PC formed around cRENPs, pRENPs, and sRENPs at different time points up to 24 h (Figure 6). At the onset, we observed the same tendency shown by the study of Piella et al.^45^ where the amount of the PC is directly dependent on the size of NPs. In our study, sRENPs were 85 nm in size (according to dynamic light scattering (DLS) measurements) and bound ∼5-fold more total protein than cRENPs or pRENPs (Figure 6A), measuring 46 and 56 nm, respectively. Furthermore, we identified the unique composition of PCs around all three different coatings bearing RENPs (Figure 6B, Supplemental Table S1). Since we have observed differences in the MDA-MB-231 uptake of cRENPs and pRENPs at the initial uptake dynamics time points (1 and 3 h), we looked for differences in their PCs (Figure 6C). Two proteins were significantly increased in cRENPs compared to pRENPs: THBS1 and adenylyl cyclase-associated protein 1 (CAP1). The former, THBS1, is an adhesive secreted lipoprotein involved in a multiplicity of biological functions, including binding to the cell surface glycoproteins and facilitating the internalization of THBS1-interacting complexes.^46^ Interaction with the low-density lipoprotein receptor-related protein (LRP) has been shown to promote THBS1 internalization via endocytosis.^47^ Moreover, MDA-MB-231 cells are known to elevate the expression of LRP receptors upon treatment with NPs up to 200 nm in size, and this altered expression results in a more efficient receptor-mediated uptake of RENPs compared to MCF-7 cells.^48^ These data correlate with enhanced cRENPs uptake by MDA-MB-231 cells and its efficient inhibition with Chlor (Figure 4). THBS1 also binds and regulates the activity of multiple growth factors,^49−51^ thus, THBS1 may facilitate the uptake of cRENPs by interacting with cell surface receptors other than LRP. Furthermore, THBS1 expression is elevated in some proliferating and tumor stroma cells;^46^ therefore, THBS1 interaction with the citrate coating may enhance the specificity when targeting tumors with RENPs. Another differentially associating PC protein, CAP1, belongs to the class of cyclase-associated proteins that couple receptor signaling to actin polymerization.^52^ CAP1 is mostly an intracellular protein but is also detected in the urine^53^ and blood serum^54^ probably due to lymphocyte cytolysis.^53^ CAP1 is often identified in the PC of various NPs;^55,56^ however, no information about the role of CAP1 on the internalization of RENPs is available to the best of our knowledge. As for PC of pRENP, an increment of two proteins, ApoA1 and F5, is observed as shown in Figure 6. However, the comparison of total PC proteomes of pRENPs and cRENPs highlights also apolipoprotein A4 (ApoA4) as overrepresented in PC of pRENPs (Supplemental Table S2). It is known that ApoA4 decreases the cellular uptake of the NPs,^57^ which matches our observation of decreased pRENPs uptake by both MDA-MB-231 and MCF-7 cells compared to cRENPs (Figures 3 and 5). We assume that both proteins ApoA4 and ApoA1 are responsible for guiding the RENPs toward internalization via CVME rather than CME. There is scarce information about the most abundant proteins found in the PC of sRENPs, A2M and F5, and their role in the internalization of RENPs. However, their abundance allows us to infer that these proteins could be the key for the macropinocytosis pathway of sRENPs in both cancer cell lines. Thus, the identification of specific components of the PC, characteristic to RENPs bearing different surface coatings, provides vital clues regarding their cellular uptake.

The combination of MCF-7 and MDA-MB-231 cell lines is a common system to explore the nuanced differences between the various types of breast cancer cells and the intracellular processes underlying these differences. MCF-7 cells are representative of chemotherapy-responsive luminal A breast carcinoma type, while MDA-MB-231 cells are representative of claudin-low subtype, included in the group of triple-negative breast carcinomas.^58^ The MDA-MB-231 cells are motile and highly invasive, and having active endocytosis is an important prerequisite.^59^ The MDA-MB-231 cells also express multiple features of the epithelial–mesenchymal transition,^60^ which is associated with the enhanced dynamics of the internalization of various receptors, for example, the epidermal growth factor (EGF) receptor.^61^ The induction of epithelial–mesenchymal transition (EMT) in the MCF-7 cell line also leads to increased EGF receptor internalization dynamics.^60,61^ The composition of the plasma membrane proteome of several breast cancer cell lines, including MCF-7 and MDA-MB-231, was described in a thorough study by Ziegler et al.^60^ However, their analysis was focused on the oncogenic properties of breast cancer cells. Aiming to identify proteins and processes underlying the difference in the uptake of RENPs, we performed our own differential analysis of cell surface proteomes of MCF-7 and MDA-MB-231 cells (Supplemental Table S4). The cell surface proteome of MCF-7 cells shows primarily the enrichment in intermediate filament keratins and catenin complexes (Table S2) that provide mechanical support for the plasma membrane and cell-to-cell contacts via cadherins and catenins, respectively. Only one functional group, endocytic vesicle proteins, related to endocytosis was enriched in MCF-7 cells in contrast to the MDA-MB-231 surface proteome. This functional group is associated with multiple biological functions responsible for cellular transport (Table S3). A cluster of clathrin-mediated transport proteins in MDA-MB-231 cells was particularly enriched consistent with our inhibitory analysis data showing the importance of the clathrin pathway to the uptake of cRENPs (Figure 4). Furthermore, having focused earlier on the THBS1 and CAP1 proteins as the most promising candidates for mediating the difference in the uptake of cRENPs and pRENPs, we analyzed the protein–protein interactions between CAP1 or THBS1 with the cell surface proteins of both cell lines *in silico*. Unfortunately, the bioinformatic analysis of CAP1 did not reveal any potential binding partners. However, being a soluble component of the extracellular matrix (ECM), THBS1 binds multiple ECM-interacting receptors that were detected in the surface proteomes of the investigated cells (Figure 7). Syndecan-1 (SDC1)^62^ and two members of the integrin family (ITGAV and ITGB5)^63^ in MCF-7 cells, and six members of the integrin family in MDA-MB-231 (ITGA1-6).^64−66^ Integrins are known to be actively internalized and recycled in both clathrin-dependent and clathrin-independent pathways, as well as during macropinocytosis.^67^ Targeting of the NPs to integrins, for example, using RGD-based peptides, is a well-known strategy to deliver the NPs in cancer cells^68^ via ITGAV and ITGA5, including MDA-MB-231 cells.^69^ Thus, as a working hypothesis, we believe that preferential association of THBS1 with the cRENPs and their exceptional internalization in MDA-MB-231 cells via CME is mediated by the interaction between THBS1 and integrins.

Endocytosis plays an important role in cancer diagnostics and therapeutics as it is the major uptake route of nanomedicines. Any and all information related to the cellular uptake of NPs have the potential to boost the effectiveness of drugs and accelerate translation of nanomedicines from the laboratory to the clinic. Our study demonstrates the fundamental RENP–cell interactions at its earliest stage, we show that the identity of the PC, contingent on the surface coating of RENPs, determines the unique pathways by which RENPs accumulate in cancer cells. Albeit these first vital data strives toward controlled and effective use of RENPs as theranostic agents, more research is still pending, such as (i) substitution of human plasma instead of a cell growth medium supplemented with FBS for the most accurate determination of the PC composition, (ii) examination of the potential of RENPs to cross the blood–brain barrier, and (iii) direct observation of RENPs’ accumulation and behavior in tissues *in vivo*.

## Conclusions

A detailed study of LiYF_4_:Yb^3+^,Tm^3+^ RENPs coated with citrate, phospholipids, or SiO_2_ (cRENPs, pRENPs, and sRENPs, respectively) conclusively demonstrated that they were biocompatible and colloidally stable in a medium containing serum proteins. In fact, the PC around the RENPs plays a major role in their stability, accumulation dynamics, and cellular uptake mechanisms. Our results showed that MDA-MB-231 cells accumulate RENPs in greater quantities than the MCF-7 cell line, especially cRENPs. Different uptake dynamics were explained by the various proteins attached to the RENPs’ surface. The proteomic and inhibitory analysis of MCF-7 and MDA-MB-231 cells presented in this work confirms the prominent upregulation of the components of CME in MDA-MB-231 cells and its role in the internalization of cRENPs. Distinctive proteins in PC around pRENPs target them via CVME. Moreover, proteins found in the PC of sRENPs activate the mechanism of macropinocytosis in both breast cancer cell lines. Finally, our results could be extrapolated to other RENPs of similar shape and size with these surface modifications; thus, aiding in systemic future *in vivo* investigations on the biodistribution and safety of RENPs, and eventually the desirable applications in various areas of nanomedicine.

## Experimental Section

A complete description of RENP synthesis, their surface modification, structural and optical characterizations, cell experiments, and proteomic analysis are provided in the Supplementary Information. Briefly, LiYF_4_:25 mol% Yb^3+^, 0.5 mol% Tm^3+^ RENPs were synthesized via a thermal decomposition method.^30,70^ Further surface modification of oleate-capped RENPs with citrate, phospholipids, and SiO_2_ was performed. Oleate-capped RENPs were characterized by X-ray powder diffraction (XRD). All RENPs were analyzed by transmission electron microscopy (TEM) and Fourier transform infrared (FTIR) spectroscopy. Upconversion spectra of all RENPs were measured upon 980 nm laser excitation (88.75 W/cm^2^). Hydrodynamic size and ζ potential of RENPs were evaluated via a dynamic light scattering (DLS) system. For the cell experiments, two human adenocarcinoma cell lines were used, MCF-7 and MDA-MB-231. Cell viability was determined by lactate dehydrogenase (LDH) and using the ADAM-MC Automatic Cell Counter. Accumulation of coated RENPs in cells was evaluated as the emission intensity of the RENPs accumulated in the cells using a 980 nm laser (118 W/cm^2^). Intracellular imaging studies were performed with a confocal laser scanning microscope. For endocytosis inhibition studies, inhibitors nystatin, chlorpromazine, nocodazole, and 5-(*N*-ethyl-*N*-isopropyl)amiloride (EIPA) were used. Proteomic analysis of the PC formed around the differently coated RENPs and the cell surface proteome of the MCF-7 and MDA-MB-231 cells were identified by high-definition liquid chromatography–mass spectrometry (LC–MS).

## Supporting Information Available

Complete description of experimental methods, proteomic data of protein corona, cell surface proteome of cancer cells, LC–MS-based protein identification and analysis of proteomic data, impact of the endocytosis inhibitors on the cell viability, XRD and FTIR spectra of RENPs, additional cellular internalization images (PDF)
Protein corona proteomics (Supplemental Table S1) (XLSX)
PC proteins with significant difference (Supplemental Table S2) (XLSX)
sRENPs protein corona pathways analysis (Supplemental Table S3) (XLSX)
Cell surface proteome (Supplemental Table S4) (XLSX)
