# Supplementary material for: Uptake of Upconverting Nanoparticles by Breast Cancer Cells: Surface Coating versus the Protein Corona
Source: ACS Appl Mater Interfaces. 2021 Aug 11;13(33):39076–87. doi: 10.1021/acsami.1c10618 (PMC8824430; doi:10.1021/acsami.1c10618)
Supplement: Supplementary file 1 — am1c10618_si_001.pdf [file am1c10618_si_001.pdf]

SUPPORTING INFORMATION

Uptake of Upconverting Nanoparticles by Breast Cancer Cells: Surface Coating versus the Protein Corona

Evelina Voronovic[abc], Artiom Skripka[d], Greta Jarockyte[ab], Marija Ger[e], Dalius Kuciauskas[e], Algirdas Kaupinis[e], Mindaugas Valius[e], Ricardas Rotomskis[af], Fiorenzo Vetrone[d], and Vitalijus Karabanovas[ac]*

[a] Biomedical Physics Laboratory of National Cancer Institute, Baublio 3B, LT-08406, Vilnius, Lithuania

[b] Life Sciences Center, Vilnius University, Sauletekio av. 7, LT-10257, Vilnius, Lithuania

[c] Department of Chemistry and Bioengineering, Vilnius Gediminas Technical University, Sauletekio av. 11, LT-10223, Vilnius, Lithuania

[d] Centre Énergie, Matériaux et Télécommunications, Institut National de la Recherche Scientifique, Université du Québec, 1650 Boul. Lionel-Boulet, Varennes, QC, J3X 1S2, Canada

[e] Institute of Biochemistry, Life Sciences Center, Vilnius University, Sauletekio av. 7, LT-10257, Vilnius, Lithuania

[f] Biophotonics group of Laser Research Centre, Vilnius University, Sauletekio av. 9, LT-10222, Vilnius, Lithuania

***Corresponding author:** vitalijus.karabanovas@nvi.lt

Table of Contents

Experimental section 3
Precursor synthesis 3
Synthesis of $LiYF_4$: Yb^{3+}, Tm^{3+} RENPs 3
RENPs' transfer to water 4
Structural characterization of RENPs 5
Optical characterization of RENPs 5
Hydrodynamic size and zeta potential characterization of RENPs 5
Colloidal stability evaluation of RENPs 6
Cell culturing 6
Cell viability assays 6
Accumulation dynamics 7
Preparation of samples for in vitro imaging 7
Imaging of RENPs in cancer cells 8
Endocytosis inhibition assays 8
Proteomic analysis of protein corona composition 9
Cell surface proteome of MCF-7 and MDA-MB-231 cells 10
LC-MS based protein identification and analysis of proteomic data 10
Statistical analysis 11
Impact of the endocytosis inhibitors on the cell viability 12
XRD and FTIR spectra of RENPs 14
Additional cellular internalization images 15
Cell surface proteome 16
Supporting Tables 17
References 18

Experimental section

Precursor synthesis

$LiYF_4$ RENPs doped with Yb^{3+} (25 mol%) and Tm^{3+} (0.5 mol%) rare-earth ions were synthesized via the previously reported thermal decomposition method[1,2].

Rare-earth (Y^{3+}, Yb^{3+} and Tm^{3+}) trifluoroacetate precursors were prepared by mixing 0.93125 mmol of Y_2O_3 (99.99+ %), 0.3125 mmol Yb_2O_3 (99.99+ %), and 0.00625 mmol Tm_2O_3 (99.99+ %) with 5 mL trifluoroacetic acid (99 %) and 5 mL of distilled water in a 100 mL three-neck round bottom flask. The mixture was then refluxed under vigorous stirring at 80 ºC until a previously turbid solution became clear, at which point the temperature was decreased to 60 ºC in order to slowly evaporate the excess trifluoroacetic acid and water. Precursors were obtained as solid dried materials and were used for the RENPs synthesis without further purification. All materials involved in the precursor synthesis were obtained from Alfa Aesar, USA, and were used without further purification.

Synthesis of $LiYF_4$: Yb^{3+}, Tm^{3+} RENPs

Dried rare-earth trifluoroacetate precursors were mixed together with 2.5 mmol of lithium trifluoroacetate (97 %, Alfa Aesar, USA), 15 mL oleic acid (90 %, Alfa Aesar, USA), 5 mL oleylamine (70 %, Sigma Aldrich, Germany) and 20 mL 1-octadecene (90 %, Alfa Aesar, USA). The solution was degassed at 110 ºC under vacuum with magnetic stirring for 30 min. After the degassing, the solution was placed under inert Ar atmosphere and the temperature was slowly raised to 330 ºC at which point the mixture was left to react for a total of 1 h.

After the reaction, solution was allowed to cool down to room temperature, maintaining the magnetic stirring and Ar atmosphere. Subsequently, the oleate-capped RENPs were precipitated with ethanol and recollected via centrifugation at 7000 RPM for 15 min. The obtained RENPs were washed twice with a mixture of hexane/acetone (1/4 v/v) and precipitated via centrifugation. Finally, the oleate-capped RENPs were re-dispersed in hexane, cyclohexane or chloroform for storage, physical characterization, and surface modification.

RENPs' transfer to water

Citrate coating of the RENPs. Citrate coated RENPs (cRENPs) were prepared via the previously reported ligand exchange procedure with some midification[3]. 50 mg of oleate-capped RENPs dispersed in 25 mL of hexane were mixed together with 25 mL of 0.2 M trisodium citrate buffer (pH 4) and left under vigorous stirring for 3 h. The two-phase (aqueous/organic) mixture was poured into the separatory funnel, and the aqueous phase containing the RENPs was isolated. The RENPs were then precipitated with acetone (1/3 v/v) via centrifugation at 7500 RPM for 30 min. The obtained pellet was re-dispersed in 25 mL of 0.2 M trisodium citrate buffer (pH 7) and left under stirring for an additional 2 h. RENPs were then precipitated with acetone (1/3 v/v) via centrifugation at 7500 RPM for 30 min, and washed twice with a mixture of water/acetone (1/3 v/v). The cRENPs were finally re-dispersed in 5 mL of distilled water and stored as is at 4 ºC for further experiments.

Phospholipid coating of the RENPs. RENPs were transferred into an aqueous environment following a modified previously reported phospholipid coating method[4]. 50 mg of oleate-capped RENPs were re-dispersed in 8 mL of chloroform together with 4.48 mg (1.6 µmol) of 1,2-dioleoyl-*sn*-glycero-3-phosphoethanolamine-N-[methoxy(polyethylene glycol)-2000] (PEG-DOPE) and 4.67 mg (6.4 µmol) of 1,2-dipalmitoleoyl-sn-glycero-3-phosphocholine (DOPC) phospholipids (Avanti Polar Lipids, Inc., USA), with a final PEG-DOPE/DOPC molar concentration ratio of 1/4. The content was lightly swirled by hand, shortly after following chloroform evaporation at 45 ºC under inert Ar atmosphere and slow magnetic stirring, resulting in a dry phospholipid-RENP film. Subsequently, distilled water (5 mL) was added in order to hydrate the film under sonication at 60 ºC temperature for 1 h. The mixture was then passed through 0.45 µm filter to remove large phospholipid and RENP-phospholipid structures, at which point the final phospholipid coated RENPs (pRENPs) dispersion was stored as is at 4 ºC for further experiments.

Silica coating of the RENPs. RENPs were rendered water dispersible via silica coating adapting the previously reported procedure[5]. 25 mg of RENPs were dispersed in 10 mL of cyclohexane and 0.1 mL Igepal (CO-520, Sigma Aldrich, Germany). After 10 min of magnetic stirring, an additional 0.4 mL Igepal together with 80 µL NH_4OH was added to the mixture and left under sonication for 20 min. The mixture was then placed under constant magnetic stirring, following the addition of 55 µL tetraethylorthosilicate (TEOS; 99+ %, Alfa Aesar, USA), and left to react for 24 h at room temperature. The silica coated RENPs

(sRENPs) were precipitated with acetone (1/3 v/v, 7500 RPM, 30 min), washed via centrifugation with pure ethanol and then an ethanol/water (1/1 v/v) mixture. Finally, the sRENPs were re-dispersed in 5 mL of distilled water and stored as is at 4 ºC for further experiments.

Structural characterization of RENPs

The crystallinity and phase of the RENPs were determined via powder X-ray diffraction (XRD) analysis on a Bruker D8 Advance Diffractometer using CuKα radiation. The morphology and size distribution of the RENPs were determined by the transmission electron microscopy (TEM, Philips Tecnai 12). The particle size was determined from TEM images using ImageJ software with a set size of 300 particles.

Fourier-transform infrared (FTIR) spectra of the synthesized oleate-capped RENPs, cRENPs, pRENPs and sRENPs were recorded with a Thermo Fisher Scientific Nicolet 6700 FTIR spectrometer. The specimens were prepared mixing together few milligrams of dried samples, or a drop of liquid samples, with KBr (FTIR grade, Alfa Aesar, USA) and pressing them into tablets.

Optical characterization of RENPs

Upconversion spectra of oleate-capped RENPs in hexane and cRENPs, pRENPs, sRENPs in water were measured at room temperature with Edinburgh Instruments spectrometer FLS920 (Edinburgh Instruments, UK) with 980 nm laser (MDL–III–980–2W, Changchun New Industries Optoelectronics Technology Co., China), laser power and power density were 710 mW and 88,75 W/cm^2, respectively.

Hydrodynamic size and zeta potential characterization of RENPs

Hydrodynamic particle size and zeta potential of cRENPs, pRENPs, and sRENPs (0.5 mg/mL concentration) were measured with particle size and zeta potential analyser ZetaPALS, using a 633 nm laser (Brookhaven Instruments Inc., Holtsville, NY, USA). The corresponding dynamic light scattering (DLS) particle size distribution was acquired 10 times without pause.

Colloidal stability evaluation of RENPs

Colloidal stability in phosphate buffered saline (PBS, Gibco, Paisley, Scotland, UK), synthetic cell culture growth medium (Dulbecco's modified eagle medium (DMEM), Gibco, Waltham, MA, USA) and DMEM supplemented with 10 % (v/v) fetal bovine serum (FBS, Gibco, Waltham, MA, USA) were measured for cRENPs, sRENPs and pRENPs. The concentration of RENPs in different sols was 1 mg/mL. Upconversion emission spectra of RENPs were measured at different times over a 192 h (8 days) period after dilution. Colloidal stability was evaluated as luminescence intensity change in time, decrease of which is correlated to the precipitation of the RENPs. Luminescence intensity is shown in % as a normalized intensity values.

Cell culturing

The MCF-7 cell line was purchased from The European Collection of Cell Cultures and the MDA-MB-231 cell line was purchased from American Type Culture Collection. Cells were cultured in DMEM complete medium: DMEM supplemented with 10% (v/v) FBS, 100 U/mL penicillin, 100 µg/mL streptomycin (all from Gibco, USA). Cells were maintained at 37 °C in a humidified atmosphere containing 5% of CO_2.

Cell viability assays

The viability of MDA-MB-231 and MCF-7 cells was tested by lactate dehydrogenase (LDH) and determined using the ADAM-MC Automatic Cell Counter. For the LDH assay MDA-MB-231 and MCF-7 cells were seeded in a 96-well plate (BD Falcon, USA) at a density of $2 \cdot 10^4$ cells/well. After 24 h, old medium was replaced with fresh medium containing 4, 40, or 400 µg/mL RENPs, while medium alone without RENPs was used as a control. Cells were then incubated in the dark for 24 h. RENPs may induce cell cytotoxicity and increase membrane permeability, as a result, cells release LDH into the cell medium. The released LDH was transferred to a new plate and detected using the LDH cytotoxicity assay (Thermo Scientific, USA) by measuring absorbance at 490 nm and 680 nm with a plate-reading absorption spectrophotometer (BioTek, USA). The detection reaction was performed according to manufacturer protocol. The obtained values of absorbance were recalculated as percentage values of viability. For another viability assay performed using automatic cell counter, cells were resuspended in 100 µL of PBS, and the total cell number together with cell viability were counted by automatic cell counter ADAM-

MC (Digital Bio, Seoul, Korea). The viability of cells and the total number of the cells was automatically assessed by ADAM-MC software.

Accumulation dynamics

The accumulation rate of cRENPs, pRENPs and sRENPs was evaluated in MCF-7 and MDA-MB-231 breast cancer cells. 40 µg/mL dose of RENPs was chosen for accumulation experiments. Cells were seeded in 12-well plates (TPP tissue culture plates, Switzerland) at a density of $1 \cdot 10^5$ cells/well and kept at 37 °C in a humidified atmosphere containing 5% of CO_2 for 24 h. Subsequently, cells were incubated with RENPs for 0.5, 1, 3, 6, 9 and 24 h under 37 °C in a humidified atmosphere containing 5% of CO_2. After incubation, the medium containing RENPs was gently removed, cells were washed three times with PBS, trypsinized with 0.25% (v/v) trypsin (Gibco, USA) and centrifuged in fresh medium at 200 *g* for 7 min.

For RENP accumulation dynamics in cells, the emission intensity of the RENPs accumulated in the cells was evaluated using an Edinburgh spectrometer FLS920 with 980 nm laser (power density 118 W/cm^2; the same parameters were used for all experiments). The emission intensity of cell suspensions was measured under constant stirring. The accumulation dynamics is determined as emission intensity at the specific time divided by cell number at that time in the suspension and plotted as emission intensity per cell.

Preparation of samples for in vitro imaging

For intracellular imaging studies, cells were seeded onto an 8-well chamber slide with removable wells (Lab-Tek, Nunc, Thermo Fisher, Denmark) with a density of $3 \cdot 10^4$ cells/chamber and subsequently incubated at 37 °C in a humidified atmosphere containing 5% of CO_2 for 24 h. For the uptake dynamics and intracellular localization evaluation of the RENPs, cells were treated with 100 µg/mL of RENPs with different coatings for 1, 3, 6 or 24 h. Before imaging cells were fixed by treating them for 15 min with a sufficient amount of 4% paraformaldehyde (Sigma-Aldrich, Germany) to cover the cells fully. After fixation, cells were stained with 10 µg/mL nuclear stain Hoechst 33258 (Sigma-Aldrich, Germany) and with 165 mM Phalloidin-Alexa 488 (Invitrogen, Thermo Fisher, US), which stains the filamentous actin of cells.

Imaging of RENPs in cancer cells

The accumulation of RENPs in cancer cells was observed using a confocal Nikon Eclipse Te2000-S C1 Plus Laser scanning microscope equipped with 405 nm (Melles Griot, USA), 488 nm (Melles Griot, USA) and 980 nm (Changchun New Industries Optoelectronics Tech. Co., Ltd., China) continuous wave lasers. The nucleus stain Hoechst 33258 was excited at 405 nm, Phalloidin-Alexa 488 was excited at 488 nm and the RENPs were excited at 980 nm. Imaging was performed using 60 x/1.4 NA oil immersion objective (Nikon, Japan). The three-channel RGB detector was used: emission of RENPs was registered in the blue channel (450/17 band pass filter), although due to better visualization red pseudo color was used in images. Fluorescence of Hoechst 33258 and Phalloidin-Alexa 488 was registered in blue (450/17) and green (545/45) channels, respectively. Image processing was performed using the Nikon EZ-C1 Bronze version 3.80 and ImageJ 1.46 software.

Endocytosis inhibition assays

MCF-7 and MDA-MB-231 cells were seeded onto 12-well plates for two days with a density of $1 \cdot 10^5$ cells/well and subsequently incubated with endocytosis inhibitors nystatin (Santa Cruz, USA), chlorpromazine (Santa Cruz, USA), nocodazole (Sigma, USA), or 5-(N-Ethyl-N-isopropyl)amiloride (EIPA) (Tocris, UK) for 1 h at concentrations listed in **Table S1**. Inhibitors were prepared in DMEM complete medium. After incubation, the inhibitor rich medium was removed, cells were washed three times with fresh medium and replaced with DMEM complete medium containing 40 µg/mL of either cRENPs, pRENPs, or sRENPs. Cells were incubated for 3 h with the RENPs in a 37 °C humidified CO_2 incubator. After incubation with RENPs, cells were washed three times with PBS, trypsinized and centrifuged at 200 *g* for 7 min. Cells were resuspended in 500 µL of PBS, counted and endocytosis inhibition was observed by measuring the emission of RENPs accumulated in cells with a FLS920 spectrometer under 980 nm laser excitation.

Additionally, accumulation of RENPs in cells after treatment with inhibitors was visualized using the confocal microscope. Both MDA-MB-231 and MCF-7 cells were seeded onto a 8-well chamber slide with removable wells (Lab-Tek, Nunc, Thermo Fisher, Denmark) with a density of $3 \cdot 10^4$ cells/chamber and subsequently incubated with RENPs at 37 °C in a humidified atmosphere containing 5% of CO_2 for 24 h. Cells were incubated with endocytosis inhibitors (nystatin, chlorpromazine, nocodazole, or EIPA) for 1 h at the

concentrations listed in **Table S1**. Cell medium with inhibitors was then replaced with DMEM complete medium containing 100 µg/mL of RENPs. Cells were incubated for 3 h with RENPs in a 37 °C humidified CO_2 incubator. After incubation cells were fixed and stained as described before.

Table S1. Endocytosis inhibitors and their concentrations used in this study

Inhibitor	Concentration	Inhibition
4 °C	-	Cellular metabolism
Nystatin	25 µg/mL	Lipid raft formation
Chlorpromazine	10 µg/mL	Clathrin-mediated endocytosis
Nocodazole	5 µg/mL	Microtubule assembly/disassembly; macropinocytosis
EIPA	3 µg/mL	Macropinocytosis

Proteomic analysis of protein corona composition

cRENPs, pRENPs or sRENPs with concentrations of 40 µg/mL were incubated in 1.3 mL of DMEM with 10% of FBS at 37ºC for 1, 3, 12 or 24 h. After the incubation RENPs were washed four times with PBS via centrifugation. Proteins were eluted from RENPs with elution buffer containing 4% sodium dodecyl sulphate (SDS), 100 mM Tris/HCl pH 7.6 and 100 mM dithiothreitol (DTT) and stored at 86ºC for further analysis.

Trypsin digestion was undertaken according to a modified filter-aided sample preparation (FASP) protocol as described by Kuciauskas et al.[6]. Briefly, proteins were diluted in 8 M urea, and loaded on protein concentrators with a polyethersulfone (PES) membrane with molecular weight cutoff of 30 kDa (Thermo Fischer Scientific, Lithuania). Following two washes with urea, proteins were alkylated with 50 mM iodoacetamide (GE Healthcare Life Sciences, MA, USA). Protein concentrators were washed twice with urea and twice with 50 mM NH_4HCO_3. Proteins were digested overnight with L-1-tosylamido-2-phenylethyl chloromethyl ketone (TPCK) treated trypsin Cat. No 20233 (Thermo Fischer Scientific, Lithuania). After overnight digestion, peptides were collected from the concentrators by centrifugation at 14000 *g* for 1 min and additionally eluted by two washes using 20% CH_3CN. The eluates were combined, acidified with 0.1% CF_3COOH and lyophilized in vacuum centrifuge. The lyophilized peptides were redissolved in 0.1% formic acid.

Cell surface proteome of MCF-7 and MDA-MB-231 cells

Surface proteins of MCF-7 or MDA-MB-231 cells were concentrated using biotin labeling. For each sample cells were grown in 6 x 10 cm diameter dishes to reach subconfluent density in DMEM medium supplemented with 10% (v/v) FBS. Cells were washed three times with ice cold PBS, pH 7.4, supplemented with 2 mM $MgCl_2$ and 1mM $CaCl_2$ (PBS/Ca/Mg) and incubated for 20 min on ice in the dark with 1 mM $NaIO_4$ in 0.1 M sodium acetate (NaOAc) and 0.15 M NaCl solution, pH 5.5 to oxidize polysaccharide groups. The reaction was then quenched by adding glycerol up to 1 mM concentration in a sample, and washing cell monolayer with ice cold PBS/Ca/Mg three times. Proteins were biotinylated for 1 h with 100 mM EZ-Link Alkoxyamine-PEG4-Biotin (Thermo Fisher Scientific, Lithuania) solution in ice cold PBS supplemented with 5% (v/v) FBS and 10 mM aniline. The cell monolayer was washed 3 times with ice cold PBS and solubilized in a lysis buffer, containing 150 mM NaCl, 1% (v/v) IGEPAL CA-630, 10 mM Tris HCl, pH 7.5 and Pierce Protease Inhibitor Tablet (EDTA-free) (Thermo Fisher Scientific, Lithuania). Lysates were cleared by centrifuging at 4 °C 22 000 *g* for 15 min and incubated for 1 h in the end-over-end shaker with 70 µL bed volume of High Capacity Streptavidin Agarose (Thermo Fisher Scientific, Lithuania) at 4 °C. Agarose resin was washed three times with lysis solution and bound proteins were eluted with 200 µL elution buffer containing 4% (w/v) 3-((3-cholamidopropyl) dimethylammonio)-1-propanesulfonate (CHAPS), 7 M urea, 2 M thiourea, 10 mM Tris-HCl, pH 8.3 and 3 mM biotin. Unbiotinylated cells were used as a negative control. The eluted proteins were digested using modified FASP protocol as described above.

LC-MS based protein identification and analysis of proteomic data

Liquid chromatographic (LC) analysis was performed in a Waters Acquity ultra performance LC system (Waters Corporation, Wilmslow, UK). Peptide separation was performed on an ACQUITY UPLC HSS T3 250 mm analytical column. Data were acquired using Synapt G2 mass spectrometer (MS) and Masslynx 4.1 software (Waters Corporation) in positive ion mode using data-independent acquisition (DIA) time (65 min). Raw data were lock mass-corrected using the doubly charged ion of [Glu1]-fibrinopeptide B (m/z 785.8426; [M+2H]2+). Raw data files were processed and searched using ProteinLynx Global SERVER (PLGS) version 3.0.1 (Waters Corporation, UK). Data was analyzed using trypsin as the cleavage protease, one missed cleavage was allowed, and fixed modification was set to carbamidomethylation of cysteines, variable modification was set to oxidation of

methionine. Minimum identification criteria included 1 fragment ions per peptide, 3 fragment ions per protein and minimum of 2 peptides per protein. The following parameters were used to generate peak lists: (i) low energy threshold was set to 135 counts, (ii) elevated energy threshold was set to 25 counts, (iii) intensity threshold was set to 750 counts. UniprotKB/SwissProt bovine and human databases (2019-09-10) were used for protein identification. Label free TOP3 (3 most intense peptides) quantification, was performed and analyzed using IsoQuant[7]. Gene ontology (GO) analysis of protein datasets was performed using Enrichr enrichment analysis server, GO Biological Process 2018 and GO Cellular Component 2018 libraries[8]. STRING database (v11)[9] was used for protein-protein interaction analysis, the search was limited only to evidence-based interactions (from experiments and databases), confidence level set to medium (0.7). Cytoscape software (v3.7.2)[10] was used for the visualization of protein interaction networks.

Statistical analysis

All the data was calculated as mean ± standard deviation (SD) from N=3 independent experiments. Number of samples, n, per certain independent experiments varied between 3 and 6, and is indicated where appropriate in figure captions of the main text. Statistical significance of differences between studied groups was assessed using a two-tailed independent Student's t-test at the 95% confidence level. Significance was represented as p-value $\leq$ 0.05.

Impact of the endocytosis inhibitors on the cell viability

In order to perform endocytosis inhibition experiments on cells, we first identified the concentration of inhibitors which does not cause adverse effects on cell health. We characterized MCF-7, MDA-MB-231 cell viability (XTT colorimetric assay) in a concentration and inhibitor type dependant manner (**Figure S1**). The cells were counted and evenly distributed in 96-well plate with $2 \cdot 10^4$ cells in each well and left for 24 hours for attachment to the bottom of the wells at 37 °C in a 5% CO_2 atmosphere. Subsequently, after removal of cell culture medium, inoculated plate was incubated for 1 hour with the fresh cell culture medium containing an inhibitor of interest at different concentrations: nystatine and chlorpromazine - 10, 25, 50 µg/mL, and nocodazole - 0.5, 1, 5 µg/mL. For each inhibitor concentration 6 wells were used, experiment repeated twice. 6 control wells were left without inhibitors as blank absorbance readings. XTT was performed according manufacturer protocol. When incubation with tetrazolium dye is finished, optical density at 490 nm was assessed using BioTek 800 TS microplate reader and average value of absorbance was determined, recalculated as percentage viability and plotted as percentage of viability against experimental concentrations of inhibitors. Results show, that only chlorpromazine at higher concentrations (25 and 50 µg/mL) drastically reduced MCF-7 and MDA-MB-231 cell viability, other inhibitors - nystatin and nocodazole - only slightly affected cell viability. From these XTT assay results we chose congruent inhibitor concentrations (shown in **Table S1**) to use for further experiments.

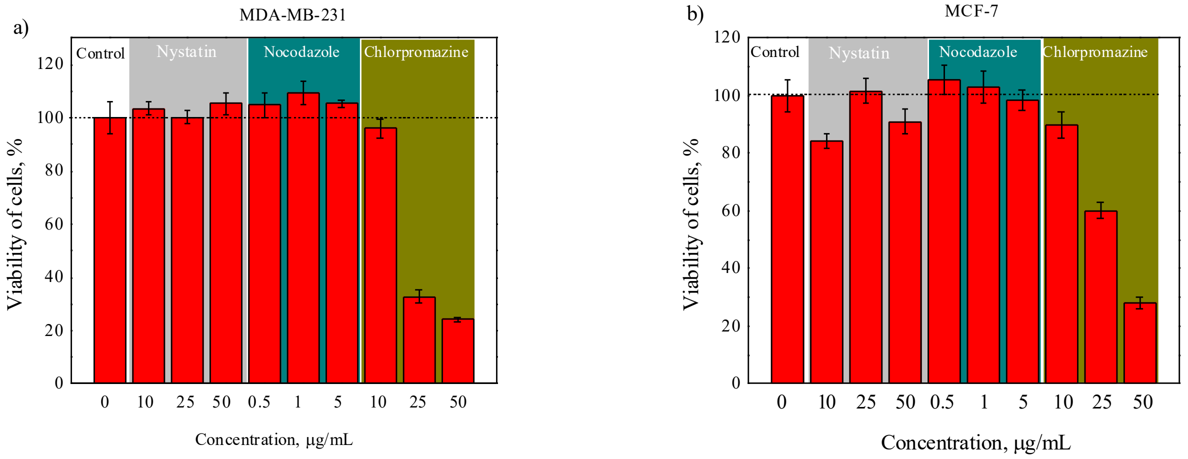

Figure S1. Viability of breast cancer cell lines: MDA-MB-231 (a), MCF-7 (b) under treatment for 1 h with various concentrations of endocytosis inhibitors: 10, 25 and 50 μg/mL of nystatin and chlorpromazine, and 0.5, 1 and 5 μg/mL nocodazole.

XRD and FTIR spectra of RENPs

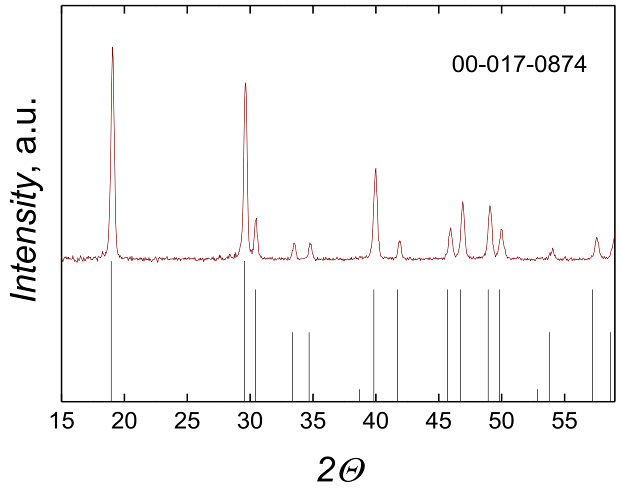

Figure S2. XRD patterns of RENPs (red line) and pure tetragonal phase (PDF #00-017-0874) (black) $LiYF_4$ nanocrystals.

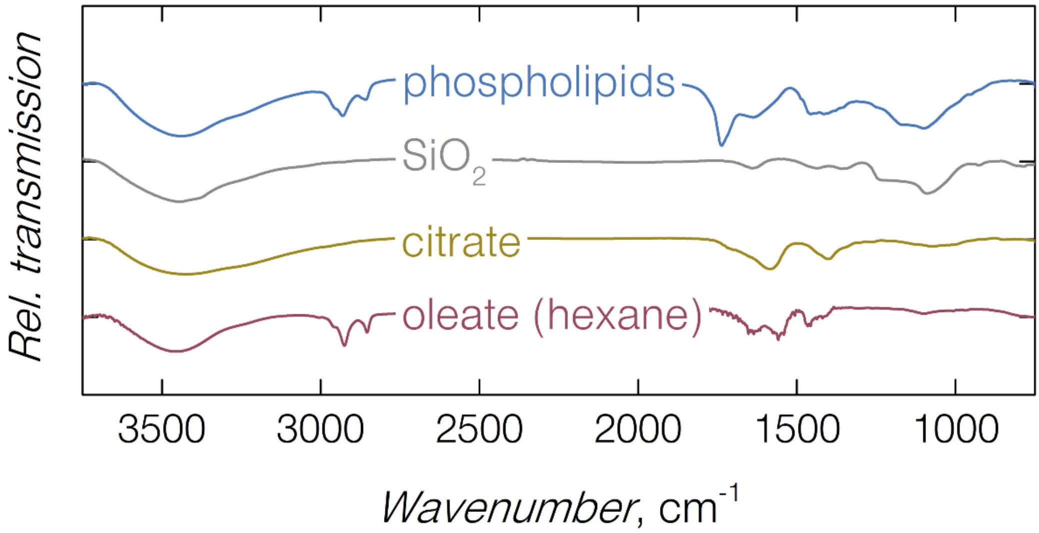

Figure S3. FTIR spectra of oleate coated parent RENPs, and those coated with citrate (cRENPs), phospholipids (pRENPs), and SiO_2 (sRENPs). Oleate-capped and pRENPs clearly showed asymmetric and symmetric stretching of $-CH_2$ groups around 2900 cm^{-1}, corresponding to their long carbon chains. Peak around 1750 cm^{-1} is attributed to C-O stretching vibrational mode of the acyl group. C=O specific vibrations around 1600 cm^{-1} indicate the presence of carboxyl groups in cRENPs. Si-O-Si, Si-O, and Si-OH vibration within the 1200-900 cm^{-1} account for the SiO_2 coating of sRENPs.

Additional cellular internalization images

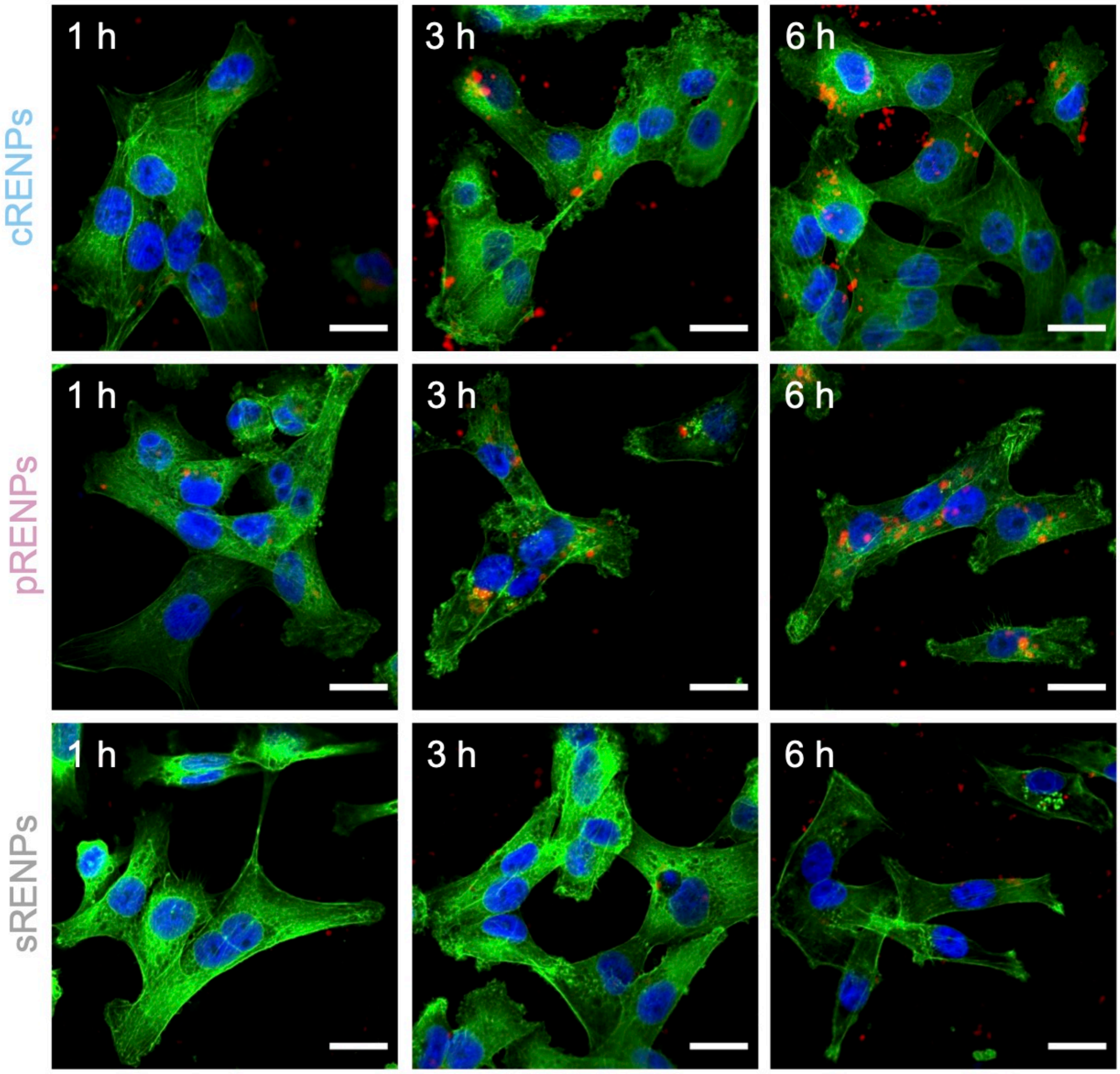

Figure S4. LSCM images of MDA-MB-231 cancer cells incubated with RENPs (100 μg/mL) at different time points: 1, 3, 6 h. Upconversion emission signal obtained under 980 nm excitation is represented by the color red in all cases. Cell nuclei were stained with Hoechst (blue)(λex = 404 nm) and F-actin was stained with Phalloidin-Alexa 488 (green) (λex = 488 nm). Scale bars in all images are 20 μm.

Cell surface proteome

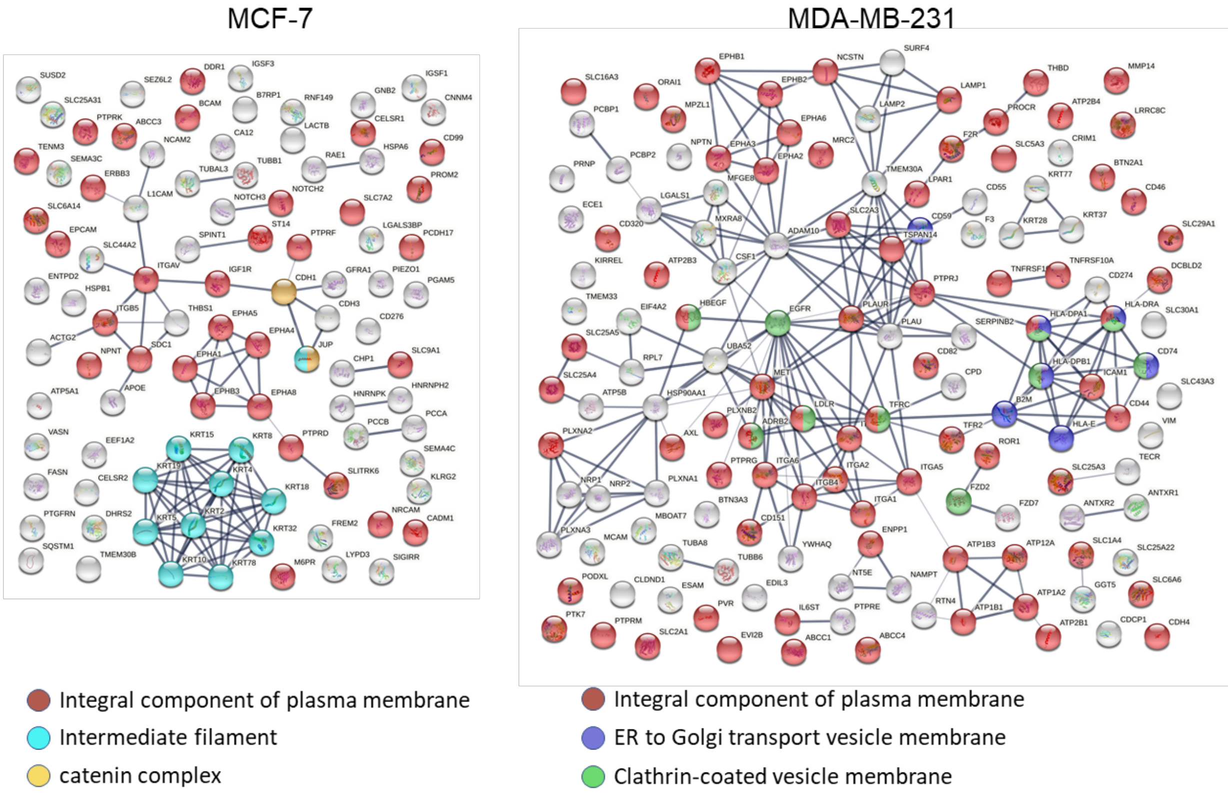

Figure S5. The analysis of the cell surface proteome of MCF-7 and MDA-MB-231 cells. Proteins overrepresented in the surface proteome of MCF-7 or MDA-MB-231 cells with the main enriched Gene Ontology categories highlighted. The protein-protein interaction networks were visualized using the STRING online resource.

Supporting Tables

Table S2. GO enrichment of MCF-7 cell surface proteins.

GO cellular component term	Gene overlap (dataset/category)	p-value of the enrichment
integral component of plasma membrane (GO:0005887)	30/1463	2.82E-12
intermediate filament (GO:0005882)	6/46	7.87E-08
polymeric cytoskeletal fiber (GO:0099513)	8/221	1.04E-05
keratin filament (GO:0045095)	3/15	4.53E-05
focal adhesion (GO:0005925)	9/356	4.96E-05
catenin complex (GO:0016342)	3/28	3.12E-04
endocytic vesicle (GO:0030139)	3/108	0.014801
axon (GO:0030424)	3/141	0.029663
cytoskeleton (GO:0005856)	6/520	0.037518
specific granule (GO:0042581)	3/160	0.040774

Table S3. GO enrichment of MDA-MB-231 cell surface proteins.

GO cellular component term	Gene overlap (dataset/category)	p-value of the enrichment
integral component of plasma membrane (GO:0005887)	64/1463	1.10E-34
focal adhesion (GO:0005925)	30/356	1.26E-23
clathrin-coated vesicle membrane (GO:0030665)	12/81	5.64E-13
ER to Golgi transport vesicle membrane (GO:0012507)	9/54	1.63E-10
integral component of lumenal side of endoplasmic reticulum membrane (GO:0071556)	7/29	1.18E-09
MHC protein complex (GO:0042611)	6/18	2.17E-09
lysosomal membrane (GO:0005765)	15/291	3.07E-09
COPII-coated ER to Golgi transport vesicle (GO:0030134)	9/75	3.39E-09
integral component of endoplasmic reticulum membrane (GO:0030176)	10/128	3.01E-08
specific granule membrane (GO:0035579)	8/91	3.05E-07

References

(1) Mahalingam, V.; Vetrone, F.; Naccache, R.; Speghini, A.; Capobianco, J. A. Colloidal Tm^{3+}/Yb^{3+}-Doped $LiYF_4$ Nanocrystals: Multiple Luminescence Spanning the UV to NIR Regions via Low-Energy Excitation. *Adv. Mater.* **2009**, *21* (40), 4025–4028.

(2) Cheng, T.; Marin, R.; Skripka, A.; Vetrone, F. Small and Bright Lithium-Based Upconverting Nanoparticles. *J. Am. Chem. Soc.* **2018**, *140* (40), 12890–12899.

(3) Naccache, R.; Chevallier, P.; Lagueux, J.; Gossuin, Y.; Laurent, S.; Vander Elst, L.; Chilian, C.; Capobianco, J. A.; Fortin, M. A. High Relaxivities and Strong Vascular Signal Enhancement for $NaGdF_4$ Nanoparticles Designed for Dual MR/Optical Imaging. *Adv. Healthc. Mater.* **2013**, *2* (11), 1478–1488.

(4) Dubertret, B.; Skourides, P.; Norris, D. J.; Noireaux, V.; Brivanlou, A. H.; Libchaber, A. In Vivo Imaging of Quantum Dots Encapsulated in Phospholipid Micelles. *Science* **2002**, *298* (5599), 1759–1762.

(5) Li, Z.; Zhang, Y.; Jiang, S. Multicolor Core/Shell-Structured Upconversion Fluorescent Nanoparticles. *Adv. Mater.* **2008**, *20* (24), 4765–4769.

(6) Kuciauskas, D.; Dreize, N.; Ger, M.; Kaupinis, A.; Zemaitis, K.; Stankevicius, V.; Suziedelis, K.; Cicenas, J.; Graves, L. M.; Valius, M. Proteomic Analysis of Breast Cancer Resistance to the Anticancer Drug RH1 Reveals the Importance of Cancer Stem Cells. *Cancers (Basel).* **2019**, *11* (7), 972.

(7) Distler, U.; Kuharev, J.; Navarro, P.; Levin, Y.; Schild, H.; Tenzer, S. Drift Time-Specific Collision Energies Enable Deep-Coverage Data-Independent Acquisition Proteomics. *Nat. Methods* **2014**, *11* (2), 167–170.

(8) Kuleshov, M. V; Jones, M. R.; Rouillard, A. D.; Fernandez, N. F.; Duan, Q.; Wang, Z.; Koplev, S.; Jenkins, S. L.; Jagodnik, K. M.; Lachmann, A.; Mcdermott, M. G.; Monteiro, C. D.; Gundersen, G. W.; Ma'ayan, A. Enrichr: A Comprehensive Gene Set Enrichment Analysis Web Server 2016 Update. *Nucleic Acids Res.* **2016**, *44* (W1), W90–W97.

(9) Szklarczyk, D.; Gable, A. L.; Lyon, D.; Junge, A.; Wyder, S.; Huerta-Cepas, J.; Simonovic, M.; Doncheva, N. T.; Morris, J. H.; Bork, P.; Jensen, L. J.; Von Mering, C. STRING V11: Protein-Protein Association Networks with Increased Coverage, Supporting Functional Discovery in Genome-Wide Experimental Datasets. *Nucleic Acids Res.* **2019**, *47* (D1), D607–D613.

(10) Shannon, P.; Markiel, A.; Ozier, O.; Baliga, N. S.; Wang, J. T.; Ramage, D.; Amin, N.; Schwikowski, B.; Ideker, T. Cytoscape: A Software Environment for Integrated Models of Biomolecular Interaction Networks. *Genome Res.* **2003**, *13* (11), 2498–2504.
